# Bisphenol A in contemporary dentistry: from material chemistry and exposure pathways to potential clinical relevance—a comprehensive narrative review

**DOI:** 10.3389/fdmed.2026.1972627

**Published:** 2026-10-06

**Authors:** Angelo Aliberti, Alexandre Luiz Souto Borges, Mirko Piscopo, Franklin Garcia-Godoy, Giovanna Orsini, Pietro Ausiello

**Affiliations:** 1 Department of Neurosciences, Reproductive Sciences and Odontostomatological Sciences, University of Naples Federico II, Naples, Italy; 2 Department of Dental Materials and Prosthodontics, Institute of Science and Technology, São Paulo State University (UNESP), São Paulo, Brazil; 3 Department of Bioscience Research, College of Dentistry, University of Tennessee Health Sciences, Memphis, TN, United States; 4 Department of Clinical Sciences and Stomatology, Polytechnic University of Marche, Ancona, Italy; 5 Interdepartmental Research Centre in Health Management and Innovation in Healthcare (CIRMIS), University of Naples Federico II, Naples, Italy

**Keywords:** biocompatibility, biological safety, bisphenol a, BPA exposure, clinical relevance, dental materials, endocrine disruptors, resin-based materials

## Abstract

**Introduction:**

Resin-based dental materials have substantially improved contemporary dental care while raising interest in their long-term biological safety. Attention has focused on bisphenol A (BPA), which may be associated with dental materials through BPA-derived monomers, residual impurities, or degradation processes. This narrative review critically synthesizes current evidence regarding BPA in dentistry, focusing on its origin and availability, biological implications, clinical relevance, and regulatory perspectives.

**Methods:**

A comprehensive narrative review was conducted through a structured search of PubMed/MEDLINE, Scopus, and Web of Science from database inception to 15 July 2026. Experimental, clinical, human biomonitoring, review, and relevant regulatory evidence published in English was integrated across defined thematic domains.

**Results:**

BPA availability from resin-based dental materials may reflect distinct processes, including elution of pre-existing impurities and BPA formation through chemical or biologically mediated degradation of susceptible BPA-derived monomers. Incomplete polymerization may increase the availability of residual resin constituents but does not itself generate free BPA, while ageing and wear may facilitate constituent release without necessarily producing BPA. Reported BPA concentrations vary according to material composition, experimental conditions, analytical methods, and sampling strategies. Human biomonitoring studies indicate that the largest post-treatment changes generally occur during early sampling periods, although their magnitude and temporal pattern vary with the procedure, treatment burden, biological matrix, sampling interval, and background environmental exposure. Available human evidence remains insufficient to establish a causal relationship between BPA exposure specifically attributable to dental materials and clinically relevant systemic adverse health outcomes. However, the absence of demonstrated long-term adverse outcomes should not be interpreted as definitive evidence of long-term safety, because longitudinal studies evaluating clinically relevant systemic outcomes remain limited.

**Conclusions:**

Current evidence supports a balanced and precautionary interpretation of BPA in dentistry. Clinical decision-making should consider the overall biological and clinical performance of dental materials while minimizing avoidable exposure whenever reasonably achievable. Analytical detection or experimental biological activity should not, in isolation, be interpreted as evidence of clinically meaningful harm; conversely, the absence of demonstrated adverse outcomes does not establish definitive long-term safety. Further longitudinal studies are required, particularly under repeated or cumulative exposure conditions and in potentially susceptible populations.

## 1 Introduction

Few innovations have influenced contemporary dentistry as profoundly as the development of resin-based dental materials (1). Their introduction has fundamentally reshaped restorative concepts by enabling minimally invasive dental treatment approaches while considerably expanding the therapeutic possibilities of modern oral healthcare (2). Continuous improvements in material composition, adhesive technologies, and polymer chemistry have progressively enhanced their mechanical resistance, esthetic integration, and long-term clinical reliability, making them indispensable across virtually every field of dentistry (3). Today, resin-based materials are routinely employed across restorative dentistry, pediatric dentistry, prosthodontics, orthodontics, endodontics, digital dentistry, and, in selected indications, oral surgery and implant dentistry (4). Alongside these unquestionable clinical advantages, increasing attention has been directed toward the long-term biological safety of these materials, particularly regarding the release of residual monomers and other potentially bioactive compounds (5).

Among these compounds, bisphenol A (BPA) has emerged as one of the most extensively investigated environmental chemicals owing to its widespread industrial use and its potential biological activity (6, 7). Widely employed in the manufacture of polycarbonate plastics and epoxy resins, BPA is now widely distributed in modern industrialized environments, resulting in continuous human exposure through dietary and non-dietary sources (8, 9). Increasing experimental, epidemiological, and toxicological evidence has suggested that BPA may interfere with endocrine regulation and several cellular signaling pathways, stimulating extensive research into its possible implications for reproductive, metabolic, neurological, cardiovascular, and immune health (10–12). These observations have progressively shifted scientific attention from the technological advantages of BPA-derived compounds toward a broader evaluation of their biological safety, prompting international regulatory agencies to continuously reassess exposure limits and risk assessment strategies (13–15).

The growing interest in BPA has inevitably extended to dentistry because several resin-based dental materials, mainly used to restore decayed teeth, are formulated using BPA-derived dimethacrylate monomers, particularly bisphenol A-glycidyl methacrylate (Bis-GMA), bisphenol A dimethacrylate (Bis-DMA), and bisphenol A ethoxylate dimethacrylate (Bis-EMA) (16). Nevertheless, an essential distinction should be emphasized. The presence of BPA-derived monomers within a dental material does not necessarily imply the intentional incorporation or direct release of free BPA. Instead, detectable BPA may originate from trace manufacturing impurities, degradation of BPA-derived monomers, incomplete polymerization, or hydrolytic and enzymatic degradation occurring after clinical placement (17, 18). Failure to recognize this distinction has frequently generated misconceptions within both the scientific literature and public debate, often leading to an oversimplified interpretation of the biological safety of contemporary resin-based dental materials.

From a clinical perspective, however, the issue extends well beyond the simple detection of BPA. Modern dentistry relies on a highly heterogeneous group of polymer-based dental materials characterized by different chemical formulations, manufacturing processes, polymerization mechanisms, and clinical indications (19). Consequently, potential BPA exposure cannot be regarded as a uniform phenomenon but rather as the result of a dynamic interaction between material composition, clinical handling, polymerization efficiency, intraoral degradation, and patient-related factors (20). Equally important, dental materials represent only one of several potential contributors to lifelong BPA exposure. Therefore, the biological significance of any BPA released from polymeric dental materials should be interpreted within the broader context of cumulative environmental exposure, toxicokinetic behavior, individual susceptibility, and the well-established therapeutic benefits that these materials have consistently demonstrated in clinical practice (21). Accordingly, the clinically relevant question is no longer whether measurable amounts of BPA may be detected following the placement of certain dental materials, but whether such exposure meaningfully contributes to systemic BPA burden or translates into clinically relevant biological effects under routine clinical conditions (22, 23).

This issue has become even more relevant as contemporary dentistry continues to evolve. The continuous introduction of novel resin formulations, BPA-free materials, ORMOCER-based systems, advanced CAD/CAM polymers, and three-dimensional printable resins has further diversified the biomaterials currently available in dentistry, creating new opportunities while simultaneously emphasizing the need for continuous evaluation of their biological performance (24–27). Providing a definitive answer remains challenging because the available evidence is remarkably heterogeneous. Current knowledge derives from analytical investigations, *in vitro* experiments, animal studies, biomonitoring analyses, and clinical research employing markedly different methodologies (28, 29). Variations in material formulations, extraction media, aging protocols, analytical techniques, biological models, sampling intervals, and outcome measures frequently limit direct comparisons between studies and contribute to the inconsistent findings reported in the literature (16, 30). Furthermore, many experimental investigations evaluate biological responses under exposure conditions that only partially reproduce the concentrations and exposure patterns encountered during routine dental treatment (22, 31). This complexity is further amplified by the widespread use of resin-containing materials throughout contemporary dental daily therapy. Potential BPA exposure is no longer confined to restorative dental procedures but may also be associated with preventive strategies in pediatric dentistry, adhesive protocols in orthodontics, prosthetic rehabilitation, digital manufacturing technologies, and several additional clinical applications (32). Accordingly, evaluating BPA from the perspective of a single discipline inevitably provides only a partial understanding of its potential clinical relevance. Likewise, assessing the biological safety of contemporary dental materials cannot rely exclusively on quantifying BPA release but should instead integrate material chemistry, exposure science, toxicological evidence, and the actual clinical conditions under which these materials are used. Such an approach is essential for distinguishing measurable chemical release from clinically meaningful biological risk and for supporting evidence-based decision-making in everyday dental practice.

Several narrative and systematic reviews have previously examined BPA in dentistry, addressing its occurrence in dental materials, potential biological effects, exposure concerns, and regulatory implications (16, 20, 33, 34). Building on this literature, the present review provides an updated synthesis that incorporates recent evidence and current regulatory guidance while placing particular emphasis on the clinical interpretation of BPA-related findings. Specifically, evidence from material chemistry, degradation and elution studies, human biomonitoring, biological investigations, and health-related studies is critically integrated to distinguish chemical detection and experimental biological activity from systemic exposure and clinically demonstrated adverse effects. This distinction is particularly relevant as resin-based technologies continue to evolve and their applications expand across restorative and pediatric dentistry, orthodontics, prosthodontics, and digital dentistry. By integrating these complementary levels of evidence, the present review aims to clarify the current clinical significance of BPA associated with dental materials, identify persistent uncertainties, and provide a contemporary framework for evidence-based interpretation and patient management (35–37).

Against this background, the aim of this comprehensive narrative review was to provide an updated and clinically oriented synthesis of the current evidence regarding bisphenol A in contemporary dentistry by integrating knowledge on dental materials, mechanisms governing BPA availability, oral exposure pathways, biological effects, and their potential clinical implications throughout contemporary dental practice. Furthermore, this review aims to provide an evidence-based appraisal of the actual clinical significance of BPA exposure associated with adhesive resin-based dental materials, identify current knowledge gaps, and discuss practical strategies to minimize patient exposure while preserving the well-established clinical effectiveness and long-term therapeutic benefits of contemporary resin-based dental materials.

## 2 Materials and methods

### 2.1 Literature search strategy

This comprehensive narrative review was developed through a structured literature search designed to identify and critically evaluate the multidisciplinary evidence regarding bisphenol A (BPA) in contemporary dentistry. The literature search was performed using PubMed/MEDLINE, Scopus, and Web of Science, selected because of their extensive coverage of biomedical, dental, materials science, toxicological, and environmental health research. Publications from database inception to July 2026 were considered. The final literature search update was performed on 15 July 2026.

The search strategy was designed to achieve broad and clinically relevant coverage of the literature concerning BPA in dentistry. Controlled vocabulary, where applicable, and free-text terms were used to explore BPA and BPA-derived monomers, dental materials, exposure pathways, biological effects, toxicological mechanisms, and the principal fields of contemporary dental practice. Representative search terms and concepts included “*bisphenol A”, “BPA”, “Bis-GMA”, “Bis-DMA”, “Bis-EMA”, “dental materials”, “resin-based materials”, “composite resin”, “dental composite”, “adhesive”, “pit and fissure sealant”, “orthodontic adhesive”, “resin cement”, “CAD/CAM resin”, “3D-printed resin”, “monomer release”, “BPA release”, “leaching”, “elution”, “polymerization”, “degree of conversion”, “hydrolysis”, “degradation”, “oral exposure”, “bioavailability”, “biomonitoring”, “saliva”, “endocrine disruption”, “estrogenic activity”, “cytotoxicity”, “genotoxicity”, “oxidative stress”, “restorative dentistry”, “pediatric dentistry”, “orthodontics”, “prosthodontics”, “endodontics”, “oral surgery”, “implant dentistry”, “periodontology”*, and “*oral medicine”*. Search terms were combined iteratively according to the specific topic being investigated, with terminology and combinations adapted to the indexing and search functions of each database. As complete database-specific search histories were not retained during the original literature search, exact query strings are not reported and were not retrospectively reconstructed.

Consistent with the narrative design of the review, the literature search was intended to support a comprehensive and critical synthesis of the available multidisciplinary evidence rather than to establish a formally enumerated set of included studies for systematic or quantitative evidence synthesis. To further broaden the evidence base, the reference lists of relevant original studies and review articles were manually examined to identify additional publications considered pertinent to the scope of the review.

Regulatory and guidance documents were identified separately from the bibliographic database searches through consultation of the official websites and document repositories of the relevant regulatory and public-health authorities cited in this review. These sources were used to identify current health-based guidance values, regulatory assessments, and recommendations relevant to BPA exposure and its interpretation in the dental context.

### 2.2 Literature selection and eligibility criteria

The literature search and identification of potentially relevant publications were conducted collaboratively by two authors (A.A. and M.P.), who subsequently evaluated a shared set of candidate publications for relevance to the scope and eligibility criteria of the review. Any differences regarding the relevance, selection, or interpretation of individual publications were resolved through discussion between the two authors and, when necessary, consultation with a third senior author (P.A.) until consensus was reached.

Given the multidisciplinary scope of the review, evidence from different levels and domains was considered, including original research articles, clinical investigations, observational studies, *in vitro* and *in vivo* experimental studies, biomonitoring analyses, analytical chemistry investigations, systematic reviews, meta-analyses, narrative reviews, consensus statements, and relevant reports issued by scientific societies or regulatory agencies. Publications were considered relevant when they addressed BPA or BPA-derived compounds in relation to dental materials, mechanisms of release or degradation, oral or systemic exposure, biological effects, toxicological mechanisms, or potential clinical implications in dentistry.

Publications primarily addressing non-dental applications of BPA without direct relevance to the objectives of the review, conference abstracts, letters to the editor, editorials without substantive scientific content, and publications unavailable in full text were not considered. Only publications in English were considered.

Within this broad methodological framework, particular emphasis was placed on evidence providing clinically or mechanistically relevant information and contributing to the interpretation of BPA chemistry, BPA-derived dental monomers, mechanisms governing BPA availability, exposure pathways, biological effects, and their potential implications across contemporary dental practice. The literature was therefore selected according to its relevance to the thematic domains of the review and its contribution to a critical and clinically meaningful interpretation of the available evidence. Accordingly, study selection involved author judgment regarding the relevance and contribution of individual publications to the defined thematic scope of the narrative synthesis. Formal screening counts were not prospectively recorded during study identification and selection and have therefore not been reconstructed retrospectively.

Because the present study was designed as a comprehensive narrative review rather than a systematic review, no formal study-level risk-of-bias assessment or methodological quality scoring system was applied. This decision also reflected the marked methodological heterogeneity of the evidence base, which encompassed analytical chemistry investigations, *in vitro* and *in vivo* experimental studies, human biomonitoring studies, observational investigations, clinical studies, reviews, and regulatory documents for which no single risk-of-bias instrument would provide a methodologically appropriate or directly comparable assessment across all evidence domains. Instead, pivotal studies were critically appraised within their respective methodological contexts according to design-specific characteristics relevant to their interpretation. For human biomonitoring and clinical studies, particular consideration was given to study design, sample size, treatment burden, biological matrix, sampling schedule, analytical methodology, potential contamination and competing exposure sources, duration of follow-up, and the distinction between exposure biomarkers and health-related outcomes. For experimental studies, interpretation considered the biological model, exposure conditions, tested concentrations, material preparation, ageing conditions, analytical methodology, and relevance to clinical exposure. Attention was also given to study-specific limitations that could influence the interpretation or generalizability of the reported findings.

In interpreting the evidence, attention was paid to the type of evidence and to the conclusions that could reasonably be supported by each study design. Mechanistic and *in vitro* studies were primarily considered for biological plausibility and material-related mechanisms; human biomonitoring studies for the characterization of internal exposure and its temporal pattern; observational human studies for potential associations between exposure and biological or health-related outcomes; and studies directly evaluating clinical outcomes for assessing the clinical significance of BPA exposure associated with dental materials. This distinction was maintained throughout the synthesis to avoid extrapolating mechanistic or exposure-based findings to clinically demonstrated adverse effects. When findings differed among studies, discrepant results were retained in the synthesis and interpreted in relation to differences in study design, population or experimental model, exposure conditions, biological matrix, analytical methodology, sampling schedule, and measured endpoints rather than being resolved through a uniform scoring system. The level of inference attributed to each study was therefore considered in relation to its design, population or experimental model, exposure scenario, biological matrix, and analytical endpoint.

Data presented in Supplementary Table 1 were extracted from the original publications by one author (A.A.) and subsequently cross-checked against the source publications by a second author (M.P.). Any discrepancies were resolved through re-examination of the relevant source publication and discussion until consensus was reached.

### 2.3 Evidence synthesis

The selected literature was critically analyzed and synthesized using an initial thematic framework defined according to the objectives and scope of the review. This framework was subsequently refined during evidence synthesis to reflect additional themes and distinctions identified across the available literature. To facilitate a comprehensive interpretation of the topic, the evidence was organized into thematic sections encompassing the chemistry of BPA and BPA-derived dental monomers, dental materials potentially associated with BPA exposure, mechanisms governing BPA release, oral exposure pathways and bioavailability, molecular mechanisms underlying BPA biological activity, clinical implications, and current approaches aimed at minimizing BPA exposure while preserving the established clinical benefits of resin-based materials.

Given the heterogeneity of the available literature, evidence was interpreted according to its methodological domain and level of clinical inference rather than being considered as a single homogeneous body of evidence. This distinction was used as an interpretative framework rather than as a formal hierarchy or grading system for the available evidence. Mechanistic and *in vitro* investigations were used primarily to characterize material-related processes, including BPA release and degradation pathways, and to establish biological plausibility through evidence concerning receptor-mediated signaling, oxidative stress, inflammatory responses, and other cellular mechanisms. Biomonitoring studies were considered primarily as evidence of exposure, providing information on the detection, magnitude, and temporal profile of BPA in biological matrices following dental procedures, but were not interpreted independently as evidence of adverse health effects. Observational human studies were evaluated for associations between exposure and biological or health-related outcomes, with consideration of potential confounding and background environmental exposure. Evidence directly evaluating clinical outcomes was considered the most clinically informative when assessing whether BPA exposure associated with dental materials translates into measurable adverse effects in patients. Accordingly, conclusions regarding clinical relevance were based on the convergence of evidence across these domains, while avoiding direct extrapolation from analytical detection or experimental biological effects to demonstrated clinical risk.

Attention was devoted to integrating evidence originating from materials science, analytical chemistry, toxicology, oral biology, and clinical dentistry to provide a critical interpretation of the available literature. Whenever discrepancies among studies were identified, the findings were interpreted considering differences in material composition, experimental design, analytical methodology, exposure conditions, biological models, and clinical applicability.

The primary objective of the evidence synthesis was to critically integrate current knowledge rather than merely describe the available literature. Accordingly, emphasis was placed on identifying areas of scientific consensus, unresolved controversies, methodological limitations, clinically relevant knowledge gaps, and future research priorities concerning the biological significance of BPA associated with contemporary dental materials.

## 3 Results

The scientific literature addressing bisphenol A (BPA) in dentistry has expanded substantially, reflecting both the continuous evolution of resin-based dental materials and the growing interest in their biological safety. Initial investigations primarily focused on identifying BPA-derived monomers incorporated into dental formulations and characterizing the chemical composition of resin-based materials (38). As analytical methodologies became increasingly sensitive and knowledge of endocrine-disrupting chemicals progressed, research gradually broadened to include BPA release under simulated and clinical oral conditions, patterns of oral exposure, biomonitoring studies, molecular mechanisms of biological activity, and their relevance to contemporary dental practice (39, 40).

Current evidence is inherently multidisciplinary, encompassing material science, analytical chemistry, toxicology, molecular biology, experimental medicine, and clinical dentistry. Consequently, the available literature includes a broad spectrum of experimental approaches, ranging from physicochemical characterization of dental materials and laboratory-based investigations to *in vitro* and *in vivo* studies, human biomonitoring analyses, and clinical research. Although these research domains have evolved independently, their integration offers a comprehensive framework for understanding the origin, release, biological activity, and clinical relevance of BPA associated with contemporary dental materials.

### 3.1 Chemical foundations of bisphenol A and BPA-derived dental monomers

Accurate interpretation of the available evidence regarding bisphenol A (BPA) associated with dental materials requires a clear understanding of the chemical relationship between BPA and the methacrylate monomers used in contemporary resin-based restorative systems (33). Despite substantial advances in dental material science, the terms BPA, BPA-derived monomers, and BPA-containing dental materials continue to be used interchangeably in parts of the scientific literature although they refer to chemically distinct entities with different molecular structures, degradation pathways, and biological implications (Figure 1). This distinction is fundamental, as it underpins the interpretation of virtually all experimental and clinical studies investigating BPA release, exposure assessment, and potential biological effects in dentistry (41).

**Figure 1:**
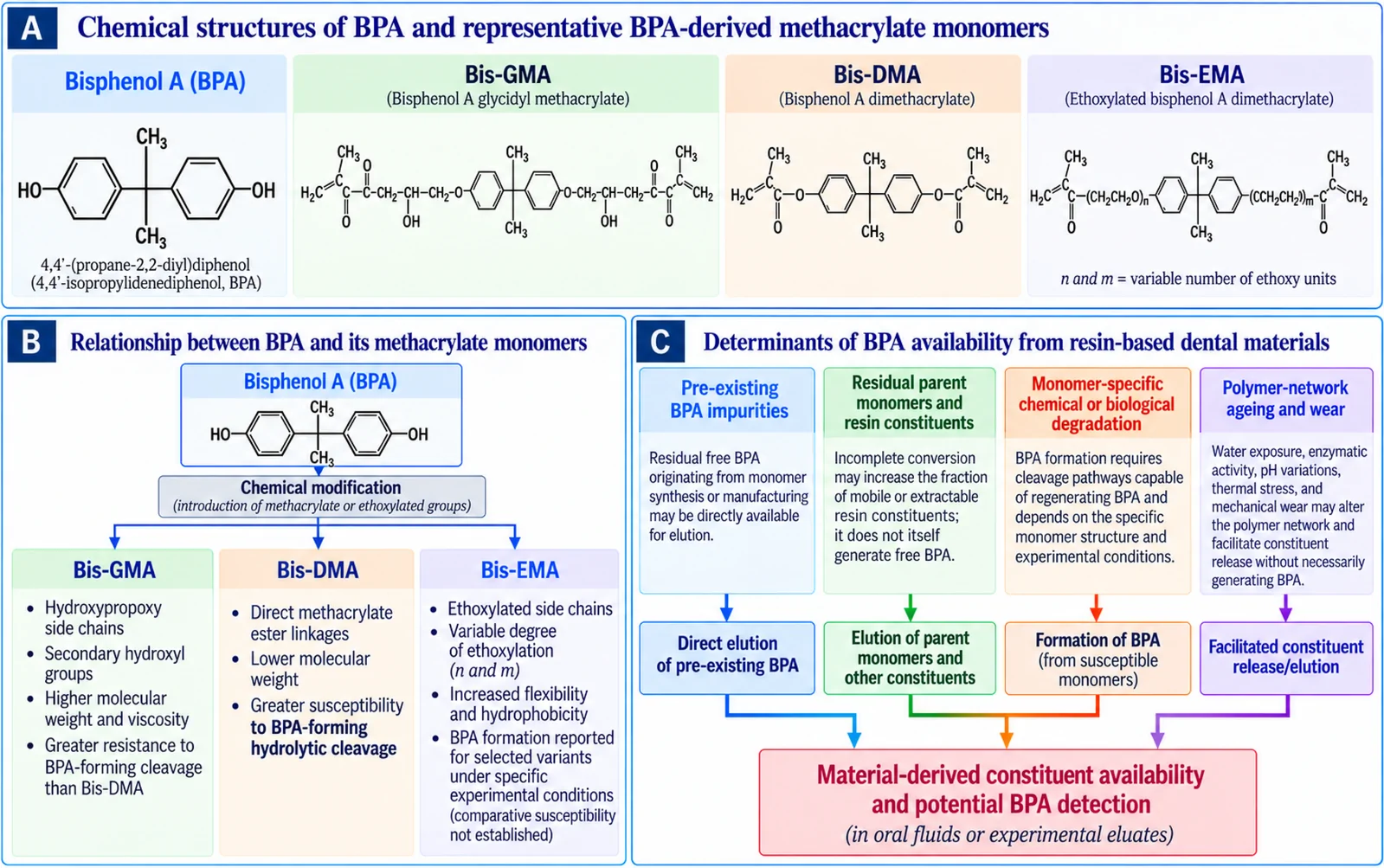
Structural relationships between bisphenol A and representative BPA-derived methacrylate monomers and determinants of BPA availability. **(A)** Chemical structures of bisphenol A (BPA) and representative BPA-derived dimethacrylate monomers used in resin-based dental materials. Bis-GMA contains hydroxypropoxy side chains with secondary hydroxyl groups and terminal methacrylate functionalities; Bis-DMA contains methacrylate ester groups directly linked through the phenolic oxygens of the BPA-derived core; and Bis-EMA is represented schematically with a variable number of ethoxy units (n and m) in its side chains. **(B)** Structural relationships between the BPA scaffold and its methacrylate derivatives, highlighting chemical features relevant to BPA-forming degradation. Bis-DMA contains ester linkages susceptible to BPA-forming hydrolytic cleavage, whereas Bis-GMA shows greater resistance to this specific cleavage pathway. Limited BPA formation has also been reported for selected Bis-EMA variants under specific experimental conditions, although their comparative susceptibility has not been established. **(C)** Conceptual distinction among pre-existing BPA impurities, elution of residual parent monomers and other resin constituents, monomer-specific degradation capable of generating BPA, and broader polymer-network ageing and wear processes that may facilitate constituent release. Incomplete polymerization may increase the availability of residual constituents but does not itself generate free BPA. Likewise, polymer-network degradation or constituent elution should not be interpreted as evidence of BPA formation unless this conversion has been analytically demonstrated. These pathways may contribute differently to material-derived constituent availability and potential BPA detection depending on material composition and experimental or clinical conditions.

Rather than being intentionally incorporated into contemporary dental materials, BPA serves principally as the chemical scaffold from which several high-molecular-weight dimethacrylate monomers have been developed. These derivatives were specifically designed to preserve the structural rigidity conferred by the bisphenol backbone while introducing polymerizable methacrylate groups capable of forming highly cross-linked polymer networks with the mechanical strength and dimensional stability required for restorative applications (42). Consequently, the performance of modern resin-based materials relies on BPA-derived monomers rather than on free BPA itself.

In contrast, TEGDMA and UDMA are not derived from BPA and may be incorporated into resin formulations to modify their physicochemical and mechanical properties (43, 44). TEGDMA is commonly used as a lower-viscosity co-monomer to regulate resin viscosity and polymerization behavior (43), whereas conventional UDMA may function as a base monomer or co-monomer depending on the formulation (44). Importantly, the absence of a BPA-derived structure in an individual monomer does not establish that the complete commercial formulation is free of BPA, Bis-GMA, or other BPA-derived constituents.

Continuous advances in resin chemistry have led to the development of a wide range of methacrylate monomers with complementary physicochemical characteristics, enabling manufacturers to tailor the performance of resin-based materials according to specific clinical indications. Among the BPA-derived monomers, bisphenol A glycidyl methacrylate (Bis-GMA) remains the principal structural monomer of most resin-based composites and adhesive systems, whereas bisphenol A dimethacrylate (Bis-DMA) and ethoxylated bisphenol A dimethacrylate (Bis-EMA) contribute additional physicochemical properties (45). In contrast, non-BPA-derived methacrylates such as TEGDMA and UDMA may fulfill different roles within the resin matrix depending on the formulation. TEGDMA commonly serves as a lower-viscosity co-monomer, whereas UDMA may constitute a principal base monomer or be combined with other methacrylates as a co-monomer (46). The structural relationships, principal physicochemical properties, and functional roles of the major methacrylate monomers currently used in restorative dentistry are summarized in Table 1.

**Table 1:** Comparative structural, physicochemical, and functional characteristics of the principal dental methacrylate monomers relevant to BPA availability.

Monomer	Key structural characteristics	Primary function in dental materials	Key physicochemical characteristics	Potential relevance for BPA availability	Typical clinical applications	Representative references
Bisphenol A (BPA)	Diphenolic aromatic compound composed of two phenolic rings linked by an isopropylidene bridge; chemical precursor of several BPA-derived dental methacrylate monomers	Industrial chemical precursor used for the synthesis of BPA-derived methacrylate monomers rather than as a functional monomer in contemporary resin-based restorative materials	Rigid aromatic structure; relatively low molecular weight compared with commonly used dental dimethacrylate monomers	Not intentionally incorporated as a functional monomer in contemporary resin-based restorative materials. When detected, BPA may originate from residual manufacturing impurities or from degradation of susceptible BPA-derived monomers	Not used as a restorative monomer	(16, 29, 33, 40)
Bis-GMA	BPA-derived dimethacrylate containing a rigid aromatic core, ether linkages, hydroxypropoxy side chains with secondary hydroxyl groups, and terminal methacrylate functionalities	Principal structural monomer providing rigidity, mechanical strength, and dimensional stability	High molecular weight and high viscosity; relatively low polymerization shrinkage; capable of forming highly cross-linked polymer networks	Shows substantially greater resistance to BPA-forming cleavage than Bis-DMA. However, trace BPA may be present as a manufacturing impurity, and very limited BPA formation has been demonstrated following specific salivary and bacterial degradation challenges. Resistance to BPA-forming cleavage should not be equated with complete resistance of the monomer or polymer network to degradation.	Resin composites, adhesive systems, resin cements, core build-up materials	(29, 47, 48)
Bis-DMA	BPA-derived dimethacrylate in which the phenolic oxygens of the BPA-derived core are directly esterified with methacrylate groups, creating hydrolytically susceptible ester linkages	Structural monomer used in selected resin formulations	Lower viscosity than Bis-GMA; comparatively greater susceptibility to hydrolytic and enzymatic cleavage	Among the commonly investigated BPA-derived dental monomers, Bis-DMA has the clearest evidence for BPA formation through hydrolytic cleavage. Cleavage of the ester linkages connecting the methacrylate groups to the phenolic oxygens of the BPA-derived core can regenerate free BPA, as demonstrated under salivary conditions.	Pit-and-fissure sealants and selected resin formulations	(31, 47, 48)
Bis-EMA	BPA-derived ethoxylated dimethacrylate with variable ethoxylated side-chain length and without the secondary hydroxyl groups characteristic of Bis-GMA	Structural co-monomer used to reduce resin viscosity and modify handling and mechanical characteristics	Lower viscosity and greater hydrophobicity than Bis-GMA; reduced water susceptibility; physicochemical behavior varies with the degree of ethoxylation and resin formulation	BPA-derived, but current evidence does not support assigning a uniform BPA-forming susceptibility to Bis-EMA as a class. Trace BPA contamination and limited BPA formation following salivary or bacterial degradation challenges have been reported for selected Bis-EMA variants under experimental conditions.	Resin composites and selected indirect resin-based restorative materials	(45, 48)
TEGDMA	Low-molecular-weight aliphatic dimethacrylate; not derived from BPA	Reactive diluent used to reduce resin viscosity and modify handling and polymerization behavior	Very low viscosity and high molecular mobility; its proportion influences degree of conversion and polymer-network characteristics	Not BPA-derived and therefore not an intrinsic chemical precursor of BPA; it may nevertheless influence overall resin-network formation and the elution behavior of resin constituents	Resin composites, adhesive systems, flowable restorative materials	(43, 45, 46)
UDMA	Urethane-based dimethacrylate; not derived from BPA	Base monomer or co-monomer, depending on the resin formulation; contributes to polymer-network formation and may also influence viscosity, conversion, and mechanical properties.	Lower viscosity than Bis-GMA; polymer-network properties depend on formulation and co-monomer composition	UDMA is not structurally derived from BPA. However, its presence in a formulation does not establish that the complete commercial material is free of BPA, Bis-GMA, or other BPA-derived constituents; this depends on the overall material composition and analytical findings.	Resin composites, bulk-fill composites, and selected CAD/CAM resin materials	(44–46)

The characteristics summarized in this table represent general structure–property relationships and should not be interpreted as invariant properties of all commercial dental materials containing the indicated monomers. Material behavior and BPA availability may vary according to monomer purity, resin composition, relative monomer proportions, degree of conversion, polymer-network characteristics, manufacturing conditions, and ageing or exposure conditions. Importantly, pre-existing BPA impurities, elution of residual parent monomers, and BPA formed through degradation of susceptible BPA-derived monomers represent chemically distinct phenomena. Resistance of a specific linkage to BPA-forming cleavage should not be interpreted as complete resistance of the monomer or polymer network to degradation.

Bis-DMA, bisphenol A dimethacrylate; Bis-EMA, ethoxylated bisphenol A dimethacrylate; Bis-GMA, bisphenol A glycidyl dimethacrylate; BPA, bisphenol A; CAD/CAM, computer-aided design/computer-aided manufacturing; TEGDMA, triethylene glycol dimethacrylate; UDMA, urethane dimethacrylate.

From the perspective of BPA exposure, however, the molecular architecture of these monomers is particularly informative because relatively small structural differences may substantially influence their degradation pathways and potential for BPA formation. Importantly, several chemically distinct processes should be differentiated when interpreting BPA-related findings. Free BPA detected from a resin-based material may represent BPA already present as a residual impurity from monomer synthesis or manufacturing; unreacted parent monomers and other residual resin constituents may elute from an incompletely polymerized network without generating BPA; and, under specific conditions, certain BPA-derived monomers may undergo chemical or biologically mediated degradation resulting in the formation of free BPA. These processes should not be considered equivalent.

Bis-GMA consists of a BPA-derived aromatic core connected to two hydroxypropoxy side chains containing secondary hydroxyl groups and terminal methacrylate functionalities. Importantly, the epoxide groups of the glycidyl precursor are opened during Bis-GMA synthesis and are therefore not present as intact epoxide rings in the resulting monomer. Bis-DMA differs structurally in that the phenolic oxygens of the BPA core are directly esterified with methacrylate groups. Cleavage of these ester linkages can regenerate the phenolic hydroxyl groups of BPA, providing a direct structural basis for BPA formation. Accordingly, Bis-DMA represents the BPA-derived dental monomer for which hydrolysis-mediated BPA formation has been most clearly demonstrated, including under salivary conditions (47).

Bis-GMA differs from Bis-DMA in this respect because the BPA-derived aromatic core is connected to the hydroxypropoxy side chains through ether linkages and shows substantially greater resistance to BPA-forming cleavage under comparable conditions (47). This greater resistance, however, should not be interpreted as evidence that BPA formation from Bis-GMA is impossible. De Nys et al. detected trace BPA already present in the tested Bis-GMA and observed a small increase in BPA following specific salivary and bacterial degradation challenges, indicating very limited BPA formation under the experimental conditions investigated (48). Bis-EMA incorporates ethoxylated side chains whose length may vary according to the degree of ethoxylation, thereby influencing its physicochemical behavior and water susceptibility (49). Limited BPA formation has also been reported for selected Bis-EMA variants following specific salivary or bacterial challenges (48); however, the available evidence does not support assigning a uniform degree of BPA-forming susceptibility to Bis-EMA as a class.

These monomer-specific degradation pathways should also be distinguished from the elution of residual constituents associated with incomplete polymerization. A lower degree of conversion may increase the fraction of mobile or extractable unreacted resin components within the polymer network, but incomplete polymerization is not itself a chemical reaction that generates free BPA. Likewise, resistance of a specific chemical bond to BPA-forming cleavage should not be equated with resistance of the entire monomer or polymer network to hydrolysis or physicochemical ageing. BPA availability therefore reflects the combined influence of manufacturing purity, pre-existing BPA impurities, residual constituent elution, monomer-specific degradation pathways, polymer-network characteristics, and subsequent chemical, enzymatic, and physicomechanical ageing processes (17, 29, 48).

Accordingly, the presence of BPA-derived monomers cannot be interpreted as evidence that a restorative material contains detectable free BPA or necessarily generates BPA during clinical use. When BPA is detected, its origin should be interpreted in relation to the specific material and experimental context, including the possible contribution of pre-existing manufacturing impurities, demonstrated monomer-specific degradation pathways, and longer-term changes in the polymer network that may facilitate constituent release. Understanding these chemical relationships is therefore not merely a prerequisite for classifying dental materials but establishes the conceptual framework through which the evidence regarding BPA release, oral exposure, toxicological mechanisms, and clinical relevance should be critically interpreted throughout this review (Figure 1).

### 3.2 BPA-containing and BPA-derived dental materials

Resin-based materials are an essential component of contemporary dentistry and are routinely used for direct and indirect restorations, adhesive procedures, resin luting, pit-and-fissure sealants, orthodontic bonding, provisional restorations, and polymer-based CAD/CAM applications (4, 50). Even if these products are often considered collectively when discussing BPA-related concerns, they encompass a chemically heterogeneous group of materials whose resin matrices differ according to their intended clinical application and proprietary formulation (38). Consequently, the relationship between dental materials and BPA is best understood according to the composition of individual products rather than broad material classifications.

Although many resin-based dental materials share common classes of methacrylate monomers, their composition varies considerably among manufacturers and among products designed for the same clinical indication. Contemporary restorative systems typically combine BPA-derived monomers, particularly Bis-GMA and Bis-EMA, with non-BPA-derived monomers such as UDMA and TEGDMA, although the relative proportions vary substantially among products (33). However, the type and relative proportion of these monomers are product-specific and cannot be reliably inferred from generic designations such as resin composite, adhesive, or resin cement (51). Even materials belonging to the same clinical category may differ substantially in their organic matrix composition, making product-specific information considerably more informative than material classification alone.

Commercial resin formulations have evolved substantially over the past decades in response to advances in material science, manufacturing technologies, and increasing attention to the biosafety of dental materials (52). Consequently, contemporary products display greater compositional diversity than earlier generations, with manufacturers adopting different combinations of conventional methacrylate monomers, alternative resin systems, and, in selected cases, formulations specifically developed to avoid BPA-derived components (53).

Pit-and-fissure sealants deserve mention because they represented the first category of dental materials to draw scientific attention to the potential presence of BPA-related compounds (53). Early investigations involving selected commercial sealants stimulated broader research into the composition of resin-based dental materials and highlighted the importance of distinguishing among different BPA-derived monomers when interpreting analytical findings (54). These observations also demonstrated that results obtained for individual products cannot be readily extrapolated to all sealants or to resin-based materials, given the considerable variability among commercial formulations.

The growing availability of products marketed as Bis-GMA-free or BPA-free has introduced additional complexity into the interpretation of material composition. Although these terms are frequently used interchangeably, they describe different chemical concepts (55). A material formulated without Bis-GMA may still contain other BPA-derived monomers, whereas the presence of a BPA-derived monomer does not necessarily indicate the intentional incorporation of free BPA (40). Likewise, restorative systems formulated with UDMA, TEGDMA, or other alternative dimethacrylates may avoid the BPA molecular scaffold while maintaining the clinical properties required for restorative applications (56). Accordingly, commercial terminology is not a reliable surrogate for chemical composition, which can only be established by considering the specific monomers included in each formulation. Overall, characterization of resin composition provides the chemical framework necessary for interpreting BPA-related findings across contemporary dental materials. However, composition alone does not determine whether free BPA becomes available under clinical conditions, underscoring the need to distinguish material formulation from material behavior.

### 3.3 Determinants of BPA availability from resin-based dental materials

The compositional characteristics represent only the starting point for understanding BPA-related exposure from dental materials. Indeed, the presence of BPA-derived monomers within a resin formulation does not necessarily imply that free BPA will become available under clinical conditions (57). Rather than representing an intrinsic property of a given material, BPA availability results from a dynamic interplay among manufacturing characteristics, polymer chemistry, physicochemical degradation, and the oral environment (16). Consequently, BPA detection reflects the outcome of multiple interacting processes rather than the direct consequence of the presence of BPA-derived monomers alone. The principal factors currently recognized to influence BPA availability from resin-based dental materials are summarized in Table 2.

**Table 2:** Principal determinants influencing BPA availability from contemporary resin-based dental materials.

Category	Determinant	Underlying mechanism	Key considerations	Representative references
Manufacturing-related factors	Residual BPA impurities and manufacturing quality	Trace amounts of BPA may remain following monomer synthesis or manufacturing processes, depending on purification procedures and quality-control standards.	Initial BPA content is product-specific and may vary considerably among commercially available materials.	(29, 48, 56)
Material-related factors	Resin matrix composition	Monomer chemistry influences hydrolytic stability, degradation pathways, water susceptibility, and the physicochemical behavior of the resin matrix.	The presence of BPA-derived monomers does not necessarily imply that free BPA will become available under clinical conditions.	(16, 31, 47, 48)
	Degree of conversion	Incomplete polymerization leaves residual unreacted resin components entrapped within the polymer network.	Lower conversion may increase the fraction of residual resin components potentially available for diffusion, elution, or subsequent degradation.	(45, 60–62)
	Cross-linked polymer network	Polymer-network architecture influences molecular mobility, diffusion, solvent penetration, and the permeability of the cured resin matrix.	Cross-link density and network heterogeneity can influence the resistance of the polymer to solvent uptake and degradation; these effects remain formulation-dependent.	(46, 61, 64, 65)
	Water sorption	Water uptake may promote polymer plasticization, swelling, and weakening of intermolecular interactions, facilitating diffusion and physicochemical degradation.	Its magnitude and consequences depend on resin composition, network characteristics, exposure time, and environmental conditions.	(48, 64, 65)
	Hydrolytic degradation	Hydrolytic cleavage of susceptible ester bonds may occur within specific monomers and polymer networks.	May contribute to BPA availability in susceptible formulations, particularly when BPA-derived structures capable of generating BPA through hydrolytic cleavage are present.	(31, 47, 58, 66, 67)
Clinical procedure-related factors	Light-curing protocol	Irradiance, exposure time, curing distance, and light-delivery conditions influence polymerization efficiency and degree of conversion.	Inadequate light exposure may reduce conversion and increase the proportion of residual resin components potentially available for elution.	(60–62)
	Restoration dimensions	Restoration thickness and geometry influence light transmission and energy delivery throughout the restorative material.	Increasing thickness may reduce polymerization efficiency in deeper regions, depending on material optical properties and the curing protocol.	(60, 62)
	Finishing and polishing procedures	Surface treatment removes or modifies superficial resin-rich and incompletely polymerized layers and alters the exposed restoration surface.	Finishing and polishing may reduce the availability of superficial residual components immediately after polymerization; the magnitude of this effect depends on the material and protocol.	(56, 73)
	Mechanical wear	Mastication, toothbrushing, and other mechanical challenges progressively modify the restoration surface during clinical service.	Progressive wear may expose deeper polymer layers and interact with chemical and hydrolytic ageing processes over time.	(63, 68, 69)
Oral environmental factors	Salivary esterases	Enzymatic cleavage of hydrolytically susceptible ester bonds may accelerate degradation of susceptible resin components.	The magnitude and biological relevance of this process depend strongly on monomer chemistry, polymer-network characteristics, and exposure conditions.	(47, 67, 70)
	Oral pH	Acidic or alkaline conditions may modify hydrolysis, water-mediated degradation, and the elution behavior of resin-based materials.	Effects are formulation- and condition-dependent and cannot be generalized across all resin-based materials.	(53, 63, 70–72)
	Thermal fluctuations	Repeated temperature changes may contribute to physicochemical ageing and changes in polymer-network behavior.	Thermal cycling should be considered as one component of multifactorial material ageing rather than as an isolated determinant of BPA availability.	(63, 68, 69)
	Dietary solvents (e.g., ethanol-containing solutions)	Organic solvents may promote polymer swelling and facilitate solvent penetration and elution of resin constituents.	Evidence derives predominantly from experimental exposure conditions; therefore, the magnitude of effects observed *in vitro* should not be directly extrapolated to clinical exposure.	(64, 70–72)
	Biofilm and prolonged aqueous exposure	Prolonged exposure to saliva, water, microbial activity, and associated enzymatic processes may contribute to progressive degradation of the polymer network.	These processes act cumulatively and interact with material composition and other environmental challenges during clinical service.	(63, 65, 67, 70)
	Intraoral aging	Progressive physicochemical alterations arise from the combined effects of time, water exposure, mechanical loading, thermal fluctuations, chemical challenges, and biological activity.	Represents the cumulative outcome of interacting degradation processes rather than a single independent mechanism of BPA availability.	(63, 68–73)

The determinants summarized in this table should not be interpreted as independent predictors of BPA availability. Their effects are strongly material- and condition-dependent and may interact according to resin composition, polymerization efficiency, manufacturing quality, ageing conditions, and the characteristics of the oral environment. Evidence derived from experimental ageing or extraction conditions should not be directly extrapolated to the magnitude of BPA exposure occurring under clinical conditions.

BPA, bisphenol A.

One potential source of detectable BPA is free BPA already present as a residual impurity originating from monomer synthesis or subsequent manufacturing processes (29, 58). Although free BPA is not intentionally incorporated into contemporary dental materials, trace amounts may remain following the industrial synthesis of BPA-derived monomers or may be introduced during subsequent manufacturing steps. The quantity of these impurities is generally low and largely dependent on the production process, purification methods, and quality control procedures adopted by individual manufacturers (49). As a result, commercially available products belonging to the same dental material category may exhibit substantially different analytical profiles despite sharing similar clinical indications, highlighting the importance of considering product-specific formulations rather than generalized material classifications (59). When interpreting BPA measurements, several chemically distinct processes should therefore be considered separately: (i) elution of free BPA already present as a manufacturing impurity; (ii) elution of unreacted parent monomers or other residual resin constituents from the polymer network; (iii) formation of BPA through demonstrated chemical or biologically mediated degradation of susceptible BPA-derived monomers; and (iv) longer-term physicochemical ageing and wear processes that may alter the polymer network and facilitate constituent release but do not, by themselves, demonstrate chemical conversion of a parent monomer into BPA. This distinction is particularly important because detection of an eluted resin constituent does not establish that BPA has been newly formed, while BPA detected after a degradation challenge must be interpreted in relation to both pre-existing contamination and any subsequent increase attributable to degradation (29, 58).

Beyond manufacturing-related impurities, the physicochemical characteristics of the polymer matrix play a central role in determining the potential availability of BPA (16). Resin-based dental materials are designed to undergo free-radical polymerization, during which methacrylate monomers are converted into a highly cross-linked three-dimensional network. However, complete conversion of all reactive methacrylate groups is not achieved during clinical polymerization, and a variable fraction of unreacted or partially reacted resin constituents may remain within the cured polymer network (60). The degree of conversion therefore influences the amount and mobility of residual resin components potentially available for diffusion, elution, or subsequent degradation (61). Importantly, incomplete polymerization should be regarded as a determinant of residual constituent availability rather than as a chemical mechanism that directly generates free BPA. Polymerization efficiency is itself affected by several variables, including resin composition, filler loading, restoration thickness, light-curing protocol, irradiation time, and the optical properties of the restorative material (62).

Unlike industrial polymers used under relatively controlled conditions, dental resin materials function within a highly dynamic oral environment characterized by continuous mechanical, chemical, thermal, and biological challenges. Following polymerization, they are exposed to saliva, fluctuating temperatures, repeated masticatory forces, dietary components, microbial biofilms, and cyclic variations in pH, all of which contribute to progressive physicochemical aging (63). Water sorption represents one of the earliest events in this process, promoting polymer plasticization, hygroscopic expansion, and gradual weakening of intermolecular interactions within the resin matrix. These phenomena facilitate the diffusion of residual low-molecular-weight compounds while increasing the susceptibility of the polymer network to chemical degradation (64, 65). The magnitude of these changes is strongly influenced by the molecular architecture of the resin matrix, with different monomer systems exhibiting distinct hydrolytic stability and aging behavior.

Among the various degradation mechanisms, hydrolysis and biologically mediated degradation have attracted particular attention because prolonged aqueous exposure and interactions with the oral environment may progressively affect the stability of resin-based polymer networks (66, 67). However, when BPA formation is specifically considered, the relevant degradation pathway depends on the chemical bonds present within individual BPA-derived monomers (31, 47, 48). In Bis-DMA, cleavage of the ester bonds directly connecting the methacrylate groups to the phenolic oxygens of the BPA-derived core can regenerate BPA, and BPA formation from Bis-DMA has been demonstrated under salivary conditions (47). Bis-GMA differs structurally because the BPA-derived aromatic core is connected to its hydroxypropoxy side chains through ether linkages and shows substantially greater resistance than Bis-DMA to BPA-forming cleavage under comparable conditions (47). This resistance to a specific BPA-forming cleavage pathway should not, however, be equated with resistance of the entire monomer or polymer network to hydrolysis or physicochemical degradation. Moreover, subsequent experimental evidence indicates that BPA formation from Bis-GMA is not necessarily absent: De Nys et al. identified pre-existing trace BPA contamination in the tested Bis-GMA and observed a small additional increase in BPA following specific salivary and bacterial degradation challenges, indicating very limited BPA formation under those experimental conditions (48). Limited BPA formation was also reported for selected Bis-EMA variants following salivary or bacterial challenge, although the available evidence does not support assigning a uniform degree of BPA-forming susceptibility to Bis-EMA as a class (48). More broadly, hydrolysis, aqueous ageing, and other physicochemical degradation processes may alter the polymer network and facilitate the diffusion or elution of resin constituents without necessarily resulting in the formation of BPA as a degradation product (66, 67). In addition to intrinsic material characteristics, several external variables may further influence BPA availability under clinically relevant conditions. Mechanical wear associated with mastication and toothbrushing, finishing and polishing procedures, repeated thermal fluctuations caused by food and beverage consumption, and long-term intraoral aging may progressively modify the superficial characteristics of resin restorations, increasing the exposure of deeper polymer layers to the oral environment (68, 69). Similarly, acidic or alkaline conditions, ethanol-containing solutions, enzymatic activity, and prolonged storage in aqueous media have all been reported to influence the degradation behavior of resin matrices under experimental conditions (70–72). However, the magnitude of these effects remains highly formulation-dependent and is strongly influenced by the experimental protocols and analytical methodologies adopted. Accordingly, evidence that a given ageing condition alters the polymer network or increases the elution of resin constituents should not, in the absence of direct analytical evidence, be interpreted as demonstrating the formation of BPA itself.

The temporal dimension of BPA availability also requires separate consideration. Although clinical biomonitoring studies generally report the largest changes in BPA concentrations during the early period following selected dental procedures, experimental elution studies indicate that material-derived BPA release may persist for considerably longer under controlled laboratory conditions. In a 52-week experimental study, De Nys et al. demonstrated prolonged BPA elution from resin-based dental composites, with the magnitude and temporal pattern of release varying according to the material and extraction conditions (73). These findings demonstrate the potential for prolonged low-level BPA release from polymerized materials under experimental conditions but should not be interpreted as evidence of sustained systemic BPA exposure in patients. Conversely, the decline or resolution of an early salivary or urinary biomarker increase does not necessarily establish that material-derived release has completely ceased, because concentrations measured in biological matrices are additionally influenced by absorption, distribution, metabolism, elimination, sampling time, and background exposure.

The marked variability reported across experimental studies reflects substantial differences in specimen preparation, polymerization protocols, extraction media, ageing procedures, analytical techniques, and sampling intervals, which frequently preclude direct comparison of BPA measurements (17, 18, 30). Consequently, the presence or absence of detectable BPA cannot be regarded as an intrinsic characteristic of a specific category of dental material but should instead be interpreted in relation to formulation chemistry, manufacturing quality, polymerization efficiency, degradation conditions, sampling strategy, and analytical methodology (56).

Ultimately, the chemical composition of a resin-based dental material represents only the starting point for understanding BPA availability. Interpretation requires distinction between pre-existing BPA impurities, elution of residual parent compounds, demonstrated BPA formation from susceptible BPA-derived monomers, and broader ageing processes that may facilitate constituent release without necessarily generating BPA. These mechanisms may interact over time, but they should not be considered chemically equivalent. Moreover, experimental evidence of prolonged material-derived elution and clinical evidence of short-term changes in biological BPA concentrations describe different aspects of exposure and should be interpreted together without directly extrapolating one to the other. The available evidence therefore supports a multifactorial and time-dependent interpretation of BPA availability rather than attribution of detected BPA to a single material characteristic or degradation mechanism (73, 74).

### 3.4 Oral exposure, systemic bioavailability, and biological fate of BPA

The presence of BPA in the oral environment following the placement of resin-based dental materials represents the initial stage of a complex pharmacokinetic process rather than evidence of systemic exposure itself. Detecting the compound in saliva does not necessarily indicate that it has reached the systemic circulation, as internal exposure depends on subsequent processes of absorption, metabolic transformation, and elimination (22). Current evidence distinguishes oral exposure, absorbed dose, and systemic bioavailability as distinct pharmacokinetic concepts when interpreting BPA associated with dental materials (23). Once BPA becomes available within the oral cavity, it may dissolve into saliva and subsequently follow different exposure pathways. A proportion of the compound is swallowed and may be absorbed through the gastrointestinal tract, whereas another fraction may undergo direct absorption across the oral mucosa. The oral mucosa has been proposed as a potential route of uptake for lipophilic compounds because of its rich vascular supply; however, its quantitative contribution after exposure to dental materials remains poorly defined (75, 76). Available pharmacokinetic data suggest that swallowing followed by gastrointestinal absorption is likely to account for most systemic exposure after restorative treatment, although the relative contribution of the different exposure routes has yet to be fully clarified (20).

Following absorption, BPA undergoes extensive phase II metabolism, predominantly through glucuronidation and, to a lesser extent, sulfation. These conjugation reactions occur mainly in the liver, with additional metabolic activity reported in the intestinal wall (77). Conversion into conjugated metabolites markedly reduces the biological activity of the parent molecule while increasing its water solubility, thereby facilitating renal excretion (78). Consequently, unconjugated BPA generally accounts for only a small proportion of total BPA detected in biological fluids after oral exposure, whereas glucuronide and sulfate conjugates constitute the predominant circulating forms. This pharmacokinetic profile explains why transient oral exposure is not necessarily accompanied by prolonged systemic availability of biologically active BPA (79).

The interpretation of human biomonitoring studies depends on both the biological matrix analysed and the timing of sample collection. Salivary BPA primarily reflects recent local exposure within the oral cavity and is strongly influenced by the interval between restorative treatment and sampling (75). Urinary BPA, in contrast, is widely used as a biomarker of recent systemic exposure because it mainly represents conjugated metabolites eliminated after absorption (80). Plasma measurements may provide additional pharmacokinetic information, although circulating concentrations are generally low and particularly susceptible to external contamination during collection, sample processing, and analytical procedures (81). Direct comparisons among studies are therefore inherently difficult, as different biological matrices reflect distinct pharmacokinetic compartments and exposure windows rather than equivalent biological conditions. Consequently, apparently conflicting findings may sometimes arise from methodological differences rather than true biological discrepancies.

Another aspect that deserves consideration is the contribution of dental materials to overall human BPA exposure. In everyday life, individuals are continuously exposed to BPA through numerous non-dental sources, including food packaging, thermal paper, drinking water, household dust, and a wide range of consumer products (81, 82). The modest and frequently transient increases in BPA concentrations reported after restorative procedures are best interpreted within the context of cumulative environmental exposure rather than being attributed exclusively to dental materials (22). Moreover, differences in analytical methodology, sampling protocols, follow-up intervals, and study design contribute substantially to the heterogeneity observed across published investigations, further limiting direct quantitative comparisons.

Exposure to BPA originating from resin-based dental materials appears to be characterized by transient oral availability, limited systemic bioavailability, extensive metabolic conjugation, and efficient urinary elimination (83). Nevertheless, the precise contribution of restorative materials to total BPA body burden remains difficult to quantify because it is influenced by background environmental exposure as well as by methodological differences among studies.

### 3.5 Molecular mechanisms underlying BPA biological activity

The biological activity of bisphenol A (BPA) results from the coordinated modulation of multiple interconnected molecular pathways rather than from a single mechanism of action (84). Although initially recognized for its weak estrogenic properties, accumulating evidence has demonstrated that BPA influences a broad spectrum of biological processes involving endocrine signaling, redox homeostasis, mitochondrial function, inflammatory regulation, and epigenetic control (85–87). These molecular responses are highly context-dependent and vary according to cell type, receptor expression profile, exposure concentration and duration, and the physiological characteristics of the target tissue (88). Consequently, BPA-induced effects arise from an integrated network of intracellular events rather than isolated signaling alterations (10).

The interaction between BPA and hormone-responsive receptors represents the best-characterized initiating event underlying its biological activity. Owing to its structural similarity to 17*β*-estradiol, BPA can interact with both classical nuclear estrogen receptors (ER*α* and ER*β*) and membrane-associated receptors, particularly the G protein-coupled estrogen receptor (GPER) (39, 89). Although its affinity for these receptors is considerably lower than that of endogenous estrogens, BPA has consistently been shown to activate both genomic and rapid non-genomic signaling pathways under experimental conditions. These receptor-mediated interactions regulate intracellular pathways involved in cell proliferation, differentiation, metabolic regulation, and adaptive cellular responses to environmental stimuli (90, 91). Experimental evidence further suggests interactions with additional nuclear receptors, including the androgen receptor (AR) and, potentially, the estrogen-related receptor gamma (ERR*γ*), although their relative biological contribution appears less clearly established than that of classical estrogen signaling (92, 93). The extensive crosstalk among these receptor systems provides the molecular basis for the wide range of cellular responses reported across experimental models.

One of the most consistently reported downstream consequences of BPA exposure is the disruption of intracellular redox homeostasis (94). Numerous *in vitro* investigations have demonstrated increased production of reactive oxygen species (ROS), accompanied by impairment of endogenous antioxidant defense systems, including reduced activity of superoxide dismutase, catalase, and glutathione peroxidase, together with depletion of intracellular glutathione reserves (95, 96). The resulting oxidative imbalance has been closely associated with mitochondrial dysfunction, characterized by alterations in mitochondrial membrane potential, impaired ATP production, reduced bioenergetic efficiency, and dysregulation of oxidative phosphorylation (97). Rather than representing independent phenomena, oxidative stress and mitochondrial dysfunction reinforce one another, establishing a self-perpetuating cycle that progressively compromises intracellular homeostasis and increases cellular susceptibility to further biological injury (98).

Evidence directly demonstrating BPA-induced biological effects in oral cell models remains comparatively limited. In human gingival fibroblasts and periodontal ligament cells, direct exposure to BPA has been associated with concentration- and time-dependent reductions in cell viability and changes in apoptosis-related gene expression (99). Direct evidence has also been reported in human gingival keratinocytes, in which low-dose BPA exposure activated estrogen receptor *β* (ER*β*) and produced comparatively modest changes in the expression of selected genes involved in epithelial biology (100). These findings demonstrate that specific oral-derived cell populations can respond to experimental BPA exposure; however, the observed effects depend on the cell model, concentration, exposure duration, and endpoint investigated. They should therefore not be generalized to other oral cell populations or interpreted as evidence that the broader spectrum of BPA-related mechanisms described in nonoral experimental systems has been directly demonstrated in oral tissues.

Inflammatory signaling represents another important component of the broader biological response to BPA. BPA-induced activation of nuclear factor-kappa B (NF-*κ*B) and related inflammatory signaling has been demonstrated experimentally in nonoral cellular models (101). Within periodontal tissues, NF-*κ*B signaling is independently recognized as an important regulator of inflammatory responses and tissue remodeling (102). Its involvement in mechanically induced orthodontic tooth movement and associated bone remodeling has also been demonstrated experimentally in the absence of BPA exposure (103). These complementary lines of evidence support the biological plausibility of NF-*κ*B signaling as a potentially relevant pathway in the oral environment but should not be interpreted as direct evidence that BPA induces NF-*κ*B activation in periodontal cells.

Complementary computational evidence has also been reported. A multidimensional network analysis integrating network toxicology, molecular docking, molecular-dynamics simulations, and single-cell transcriptomic data identified several candidate molecular targets potentially linking BPA exposure with oral squamous cell carcinoma-related pathways (104). These findings are hypothesis-generating and should not be interpreted as direct experimental evidence of BPA-induced effects in oral cells.

Beyond their immediate effects on intracellular signaling, BPA-induced molecular alterations may also influence long-term regulation of gene expression through epigenetic mechanisms. Available evidence indicates that BPA exposure can modify DNA methylation patterns, alter histone acetylation and methylation, and regulate the expression of multiple microRNAs involved in cellular proliferation, differentiation, inflammatory responses, oxidative stress regulation, and tissue remodeling (105). Unlike genetic mutations, these epigenetic modifications do not alter the nucleotide sequence but instead regulate transcriptional activity, thereby influencing cellular plasticity and long-term adaptive responses. Although the specific molecular targets differ among experimental systems, epigenetic dysregulation is increasingly recognized as a component of BPA biological activity and may contribute to the persistence of cellular alterations after the initial exposure has ceased (106). However, direct evidence demonstrating comparable BPA-induced epigenetic alterations in oral cell populations remains limited. The relevance of these mechanisms to oral tissues should therefore currently be regarded primarily as mechanistic plausibility rather than as an established oral-cell response.

Current evidence supports the concept that receptor-mediated signaling, oxidative stress, mitochondrial dysfunction, inflammatory activation, and epigenetic regulation constitute an interconnected network of mechanisms involved in the biological activity of BPA across different experimental systems (39). However, the strength and directness of the evidence supporting these mechanisms in oral cells vary considerably. Direct experimental evidence in oral-derived cells is currently limited to a comparatively small number of models and biological endpoints, whereas other potentially relevant mechanisms are supported predominantly by BPA experiments conducted in nonoral models, studies demonstrating the relevance of individual pathways in oral tissues without BPA exposure, or computational and predictive analyses. These different lines of evidence should not be considered equivalent. Rather, taken together, they support the biological plausibility of BPA-responsive pathways potentially relevant to the oral environment while highlighting important gaps in direct oral-cell evidence. Moreover, the translational significance of these mechanistic findings remains limited by the heterogeneity of experimental models and exposure conditions. BPA concentrations, exposure durations, and culture conditions employed in experimental studies may not reproduce the magnitude, temporal pattern, metabolism, or clearance associated with dental exposure in humans; consequently, these findings should primarily be interpreted as evidence of biological plausibility rather than as quantitative evidence of clinical risk.

Table 3 summarizes the principal studies informing these mechanistic relationships and explicitly distinguishes direct experimental evidence of BPA effects in oral cells from BPA experiments conducted in nonoral models, BPA-independent evidence concerning pathway relevance in oral tissues, and computational or predictive evidence.

**Table 3:** Experimental and mechanistic evidence linking BPA-related biological pathways to oral cells and tissues.

Evidence category	Model	Tested compound/exposure	Concentration	Exposure time	Endpoint and main finding	Evidence status	Ref.
Estrogen-receptor signaling	Human gingival keratinocytes	BPA	11.4 nM, 0.16 μM, 1.3 μM	6 h, 24 h, 4 d	ER*β* activation was detected across the tested concentrations and time points, whereas modulation of keratinocyte-related gene expression was generally modest	Direct oral-cell BPA evidence	Burkhardt et al., 2021 (100)
Cell viability and apoptosis-related signaling	Human gingival fibroblasts	BPA solutions	0, 0.001, 0.01, 0.1 ppm for viability; 0, 0.001, 0.01 ppm for gene-expression analysis	0.5, 1, 6, 24 h for viability; 6 h for gene expression	Reduced cell viability following BPA exposure; BAX and CASP8 expression increased in a dose-dependent manner	Direct oral-cell BPA evidence	Jung et al., 2023 (99)
Cell viability and apoptosis-related signaling	Human periodontal ligament cells	BPA solutions	0, 0.001, 0.01, 0.1 ppm for viability; 0, 0.001, 0.01 ppm for gene-expression analysis	0.5, 1, 6, 24 h for viability; 6 h for gene expression	Reduced cell viability following BPA exposure; CASP10 expression differed significantly among BPA exposure conditions	Direct oral-cell BPA evidence	Jung et al., 2023 (99)
NF-*κ*B inflammatory signaling	MCF10A non-transformed human mammary epithelial cells	BPA	10 nM	24 h	Transcriptomic enrichment of inflammatory signaling, increased NF-κB phosphorylation and nuclear translocation, and enhanced cell motility; NF-κB inhibition attenuated BPA-induced effects	Nonoral BPA experimental evidence	Citarella et al., 2025 (101)
NF-κB signaling in periodontal tissue remodeling	Transgenic and wild-type mice; NF-κB modulation targeted to osteoblast-lineage cells and PDL fibroblasts during orthodontic tooth movement	No BPA exposure; 10–12 g orthodontic force	N/A	5 or 12 d	Orthodontic force induced NF-κB activation; inhibition of NF-κB reduced tooth movement, osteoclastogenesis and RANKL expression	Oral/periodontal pathway evidence, not BPA-specific	Jeon et al., 2021 (103)
Potential OSCC-related molecular pathways	Network toxicology, molecular docking, molecular-dynamics simulations and single-cell transcriptomic datasets	BPA evaluated computationally	N/A	N/A	PTGS2, HSP90AA1 and KEAP1 emerged as candidate targets; docking and molecular-dynamics analyses predicted BPA–target interactions, while single-cell data characterized target expression in OSCC cell populations	Computational/predictive evidence; not direct oral-cell BPA exposure	Huang et al., 2025 (104)

Evidence status was classified according to the experimental context of each study. “Direct oral-cell BPA evidence” indicates experimental exposure of oral-derived cells to BPA with direct measurement of the reported biological endpoint. “Nonoral BPA experimental evidence” indicates experimental BPA exposure in a nonoral cellular model. “Oral/periodontal pathway evidence, not BPA-specific” indicates evidence demonstrating the biological relevance of the pathway in oral or periodontal tissues without experimental BPA exposure. “Computational/predictive evidence” indicates associations derived from network-based, molecular docking, molecular-dynamics, transcriptomic, or other computational analyses rather than direct experimental BPA exposure of oral cells. Findings derived from nonoral models, BPA-independent oral studies, or computational analyses should not be interpreted as direct evidence of BPA-induced effects in oral cells. N/A indicates that the corresponding concentration or exposure time was not applicable because the study did not involve experimental BPA exposure of the indicated oral model.

BAX, BCL2-associated X protein; BPA, bisphenol A; CASP8, caspase 8; CASP10, caspase 10; ERβ, estrogen receptor beta; HSP90AA1, heat shock protein 90 alpha family class A member 1; KEAP1, Kelch-like ECH-associated protein 1; N/A, not applicable; NF-κB, nuclear factor-kappa B; OSCC, oral squamous cell carcinoma; PDL, periodontal ligament; PTGS2, prostaglandin-endoperoxide synthase 2; RANKL, receptor activator of nuclear factor-kappa B ligand.

### 3.6 BPA exposure across contemporary dental practice

The increasing use of resin-based dental materials has progressively broadened the scope of BPA-related research within dentistry (31). Initially centered on direct restorative materials, subsequent investigations have progressively expanded to encompass multiple clinical applications involving polymer-based dental materials, including preventive, orthodontic, prosthodontic, and digitally manufactured devices (16). Accordingly, the available literature now addresses a wide range of exposure scenarios, biological matrices, and analytical approaches, reflecting the extensive integration of resin-based materials into contemporary dental practice.

Direct resin-based restorative procedures constitute the most extensively investigated dental source of BPA exposure (18, 29). Contemporary composite resins encompass chemically heterogeneous formulations, and experimental studies have reported substantial product-dependent variability in BPA availability following polymerization (17, 58, 72, 74). Reported concentrations vary considerably according to resin composition, degree of conversion, ageing conditions, extraction protocols, and analytical methodology. Accordingly, the available evidence supports a material- and condition-dependent exposure profile rather than a uniform behavior applicable to all resin-based restorative materials (18).

Adhesive systems have likewise received considerable attention because they represent the first resin-based material placed in direct contact with enamel, dentin, and oral fluids during adhesive restorative procedures. Like restorative composites, contemporary bonding agents may contain BPA-derived monomers, and several investigations have evaluated the availability of BPA and related compounds following polymerization (107). The published literature demonstrates substantial variability among adhesive formulations, primarily reflecting differences in chemical composition, polymerization efficiency, filler content, and analytical sensitivity (108, 109). Consequently, currently available evidence does not support uniform BPA availability across all adhesive systems but instead indicates formulation-specific behavior that closely parallels the heterogeneity observed for restorative composites.

Clinical biomonitoring studies have evaluated BPA concentrations in saliva and urine following resin-based restorative procedures (23, 83). Saliva represents the biological matrix most extensively investigated, with increases in BPA concentrations most frequently reported during the early post-treatment period (23, 83). Urinary findings have been less consistent, with prospective studies reporting either short-term post-treatment increases or no clear treatment-related increase depending on the restorative procedure, study population, and sampling schedule (23, 80). Despite marked heterogeneity in restorative protocols, sampling intervals, analytical techniques, and study populations, the immediate post-treatment period is the interval during which increases in salivary BPA concentrations have been most consistently observed (110). Concentrations measured in biological matrices generally decline during subsequent follow-up; however, this temporal pattern should be interpreted as a biomonitoring observation rather than as evidence that material-derived BPA release has completely ceased. Experimental studies demonstrating longer-term elution under controlled conditions provide complementary information on material release but cannot, in turn, be interpreted as evidence of sustained systemic exposure in patients (73).

Several investigations have examined procedural and material-related factors that may influence the availability of residual constituents following restorative treatment. Attention has been directed toward the superficial oxygen-inhibited layer, which contains incompletely polymerized resin components and may contribute to the initial availability of resin-derived constituents (56). Removal or modification of this superficial layer through appropriate surface treatment has therefore been proposed as a potential means of limiting the availability of residual surface components. However, this rationale should be distinguished from direct evidence of reduced BPA exposure, since the available evidence does not establish that finishing or polishing produces a corresponding reduction in systemic BPA dose or BPA-related health outcomes. Separately, experimental degradation and elution studies have demonstrated that BPA availability from polymerized resin-based materials may vary according to material composition, ageing conditions, and duration of observation (73, 74). These laboratory findings provide information on material-derived BPA elution and degradation behavior but should not be interpreted as evidence that finishing or polishing reduces BPA exposure in patients.

Pit-and-fissure sealants constitute another major area of BPA-related research and represent one of the earliest resin-based materials investigated in dentistry (111). *In vivo* studies have demonstrated that BPA may be detected in saliva shortly after sealant placement, whereas serum concentrations are generally negligible or undetectable (112). Experimental findings, however, have varied substantially among commercial products, with BPA identified in eluates from certain sealants but not from others, underscoring important formulation-dependent differences (53). Similar variability has also been reported for BPA-derived monomers and other residual resin constituents, further emphasizing the influence of material composition, polymer chemistry, and curing characteristics on analytical outcomes.

Because resin-based sealants are extensively used during childhood, numerous investigations have specifically evaluated BPA exposure in pediatric populations (113, 114). Most of these studies have relied on salivary and urinary biomonitoring following preventive or restorative dental procedures involving sealants and resin-based composites commonly used in pediatric dentistry (115–117). Although study design, sampling protocols, analytical methodology, and follow-up intervals differ substantially among investigations, the existing literature consistently indicates that pediatric findings primarily reflect BPA exposure associated with the clinical use of resin-based restorative materials rather than the existence of material formulations specifically developed for children (114). Accordingly, the available pediatric evidence primarily reflects restorative resin-based dentistry rather than a distinct category of BPA exposure.

Comparable research has subsequently extended to orthodontic materials, which currently represent the second major area of BPA-related investigation in dentistry (118). Resin-based bonding agents used for fixed appliances, adhesive systems, bonded retainers, polycarbonate brackets, and, more recently, thermoplastic aligners and removable retainers have all been evaluated as potential sources of BPA and other BPA-derived compounds (119). Experimental observations indicate that their availability varies considerably according to polymer composition, curing conditions, ageing protocols, extraction media, and analytical methodology (21, 120–123). Clinical reports concerning fixed orthodontic appliances have evaluated salivary BPA concentrations following bracket bonding and debonding procedures, with transient increases reported during the early post-treatment period (124). More recently, attention has expanded to clear aligner systems and thermoplastic retention appliances, which represent distinct exposure scenarios because of differences in material composition, manufacturing processes, and patterns of intraoral contact. Salivary BPA exposure has also been investigated in patients undergoing clear aligner treatment (125). Although several investigations of thermoplastic orthodontic materials have reported minimal or undetectable BPA availability, measurable concentrations have occasionally been identified under specific experimental or clinical conditions (126–128). At present, differences in polymer chemistry, manufacturing technologies, post-processing procedures, and analytical approaches preclude the identification of a consistent exposure profile applicable to all orthodontic materials.

Recent advances in prosthodontics and digital dentistry have further expanded BPA-related research to indirect resin-based materials. Current investigations include resin cements, provisional restorative materials, CAD–CAM composite blocks, and additively manufactured polymers employed for provisional restorations, occlusal splints, surgical guides, denture bases, and other prosthodontic applications (129–131). Compared with conventional restorative composites and sealants, however, the published evidence remains considerably less extensive. Most published studies have primarily evaluated the release of residual methacrylate monomers and other organic constituents rather than unconjugated BPA itself (132, 133). When specifically investigated, BPA findings have varied according to the material and experimental conditions. In some cured 3D-printed materials, BPA was not detected under the analytical conditions employed (99), whereas other investigations have demonstrated the elution of resin constituents from digitally manufactured materials under specific experimental conditions (134). Such nondetection should be interpreted in relation to the analytical method and its detection limits and should not be considered equivalent to a quantified low BPA concentration or evidence of the complete absence of BPA release. Consequently, current knowledge remains insufficient to define consistent exposure patterns across the rapidly expanding range of digitally manufactured resin-based dental materials.

Beyond patient-related exposure, increasing attention has also been directed toward potential occupational exposure associated with the routine handling of resin-based dental materials. Throughout restorative, orthodontic, prosthodontic, and laboratory procedures, dentists, dental assistants, and dental technicians repeatedly manipulate uncured resin systems during dispensing, placement, finishing, polishing, adjustment, repair, and removal of polymer-based restorations and appliances (135). Additional opportunities for exposure may arise during grinding, trimming, polishing, and other laboratory procedures that generate resin-containing particulate matter, as well as during digital manufacturing workflows involving printable polymeric materials (136). Nevertheless, compared with the extensive literature evaluating patient exposure, relatively few studies have specifically quantified BPA exposure among oral healthcare professionals (137, 138). Consequently, the available evidence is derived predominantly from the chemical characterization of dental materials and the identification of BPA potential exposure pathways, whereas dedicated occupational biomonitoring investigations involving dental personnel remain limited.

Overall, the available literature demonstrates that BPA exposure has been investigated across a broad spectrum of contemporary dental applications, with the strongest body of evidence concerning direct resin-based restorations, adhesive systems, pit-and-fissure sealants, and orthodontic materials. By contrast, substantially fewer data are currently available for indirect restorative polymers, digitally manufactured resin-based devices, and occupational exposure in dental professionals. Considerable heterogeneity also persists regarding material composition, specimen preparation, ageing conditions, extraction protocols, biological matrices, analytical methodologies, sampling schedules, and outcome reporting. Therefore, the published findings are most appropriately interpreted according to the specific material, clinical procedure, and exposure scenario under investigation rather than being extrapolated across all resin-based dental applications.

### 3.7 Clinical risk assessment and human biomonitoring

Clinical risk assessment of BPA associated with dental materials relies on the integration of human biomonitoring data, toxicokinetic evidence, and toxicological reference values established by regulatory authorities (139). Clinical investigations have predominantly focused on quantifying BPA in biological matrices following dental procedures, thereby providing information on human exposure under routine clinical conditions (140). Because direct quantification of the absorbed systemic dose attributable exclusively to dental materials remains methodologically challenging, clinical risk assessment in dentistry has relied primarily on biomonitoring investigations evaluating BPA concentrations in saliva and urine (16).

Human biomonitoring studies have mainly assessed BPA concentrations in saliva and urine following the placement of resin-based restorations, adhesive systems, and pit-and-fissure sealants (83). Saliva represents the biological matrix most frequently investigated because it reflects direct contact between resin-based materials and the oral environment immediately after treatment (141). Across the published literature, BPA concentrations generally increase during the early post-treatment period, with the highest levels detected within minutes or the first hours following material placement. Subsequently, salivary concentrations generally decline during follow-up. In the prospective study by Berge et al., the marked increase observed shortly after restorative treatment decreased substantially within 24 hours and approached pretreatment concentrations by one week (23). Urinary biomonitoring has shown a less consistent temporal pattern. Prospective studies have reported either transient post-treatment increases or no clear treatment-related increase, depending on the restorative procedure, study population, and timing of sample collection (23, 80). Cross-sectional evidence should be interpreted separately. In the NHANES analysis by McKinney et al., urinary BPA concentrations were evaluated in relation to the presence and number of existing dental sealants and restorations rather than through within-person measurements before and after treatment; consequently, this study informs population-level associations but cannot define the temporal course of BPA exposure following a dental procedure (142). The reported findings vary considerably among studies owing to differences in study design, sampling protocols, restorative procedures, analytical methodology, and background environmental exposure (143).

The magnitude and temporal pattern of BPA detected after dental treatment should therefore be interpreted on a study-specific basis rather than summarized using a single exposure estimate. Berge et al. evaluated 20 adults undergoing treatment with polymer-based restorative materials and collected saliva and urine before treatment and at defined post-treatment intervals (23). Mean salivary BPA increased from 0.11 ng/mL before treatment to 385 ng/mL at 10 min and subsequently declined to 88.2 ng/mL at 1 h, 1.85 ng/mL at 24 h, and 0.25 ng/mL at 1 week. In contrast, density-adjusted urinary BPA concentrations were 1.41 ng/mL before treatment, 1.17 ng/mL at 1 h, 1.53 ng/mL at 24 h, and 1.99 ng/mL at 1 week, with no statistically significant post-treatment change. These contrasting patterns illustrate that salivary and urinary BPA measurements represent different exposure compartments and temporal windows and should not be interpreted interchangeably. The study also documented substantial interindividual variability and identified other potential sources of BPA exposure, including the handling of thermal-paper receipts.

A larger study by Kingman et al. included 172 participants receiving resin-based composite restorations, with BPA measurements available in saliva from 151 participants and in urine from 171 participants (83). Salivary BPA concentrations increased within the first hour after restoration placement and returned to pre-restoration levels within approximately 8 h. Urinary BPA followed a different temporal pattern, with a 43% increase relative to pre-restoration concentrations reported during the 9–30 h post-treatment interval. These findings further demonstrate that the timing and magnitude of post-treatment changes depend on the biological matrix and sampling interval. Accordingly, the term “transient” should be interpreted in relation to the specific procedure, matrix, and observation period investigated rather than as a uniform characteristic of BPA exposure following all resin-based dental procedures.

In children and adolescents, Maserejian et al. followed 91 participants aged 3–17 years before and approximately 1 day, 14 days, and 6 months after placement of Bis-GMA–based composite restorations (80). Participants received a mean of 1.4 ± 1.0 composite-restored surfaces at the first treatment visit and 2.3 ± 1.6 surfaces cumulatively during the study. The mean change in urinary BPA concentration from pretreatment to the following day was 1.71 ± 9.94 ng/mL, indicating substantial interindividual variability. In the primary analysis, a greater number of composite-restored surfaces was associated with a greater next-day increase in urinary BPA. However, one participant who received eight restored surfaces in a single visit had a next-day urinary BPA concentration of 81.8 ng/mL. After exclusion of this participant, the mean next-day change decreased to 0.87 ± 5.98 ng/mL and the association between treatment burden and next-day urinary BPA was attenuated and no longer statistically significant. No corresponding association between treatment burden and urinary BPA was observed at approximately 14 days or 6 months. These findings indicate that the early post-treatment association was sensitive to treatment burden and influential observations and should therefore not be interpreted as evidence of a uniform increase in urinary BPA following composite restoration placement.

The interpretation of these biomonitoring findings should also consider the pharmacokinetic characteristics of BPA. Following oral exposure, absorbed BPA undergoes extensive first-pass metabolism, predominantly through glucuronidation and sulfation, generating conjugated metabolites that are subsequently eliminated in urine (144). Consequently, unconjugated BPA generally represents only a minor proportion of circulating BPA following oral exposure (79). Human pharmacokinetic studies indicate relatively rapid urinary elimination of BPA following systemic exposure (79). Importantly, however, these general pharmacokinetic data should not be interpreted as direct evidence of the magnitude or time course of systemic BPA exposure specifically attributable to dental treatment.

Regulatory risk assessment has evolved considerably over recent years. Earlier investigations evaluating BPA exposure associated with dental materials generally interpreted their findings according to the tolerable daily intake values established before 2023. Following a comprehensive reassessment of the available toxicological evidence, the European Food Safety Authority (EFSA) established a tolerable daily intake (TDI) of 0.2 ng/kg body weight/day, representing a 20,000-fold reduction from the temporary TDI of 4 μg/kg body weight/day established in 2015 (36). The revised TDI was primarily derived from evidence concerning immunological effects, with an increase in the percentage of Th17 cells in the spleen identified as the critical effect for risk characterization (36). Importantly, this health-based guidance value was developed in the context of chronic daily oral exposure and was not specifically designed to assess short-term or episodic exposure associated with dental procedures (145). Since many clinical studies investigating BPA exposure from dental materials were conducted before publication of the updated EFSA Scientific Opinion, their findings were interpreted according to the toxicological reference values applicable at the time of investigation. Moreover, the TDI represents an external oral-intake benchmark expressed on a body-weight and daily-intake basis. Accordingly, BPA concentrations measured in saliva, urine, or plasma are not directly comparable with the TDI, because concentrations in biological matrices do not themselves represent an ingested dose. Similarly, comparison of estimated absorbed or internal doses with an external oral-intake benchmark requires both quantities to be placed on a comparable basis using an appropriately justified pharmacokinetic or exposure model. In the absence of such modeling, biomonitoring measurements represent indicators of exposure rather than direct measures of intake relative to the TDI.

Pediatric populations have received particular attention in the literature owing to the widespread use of resin-based sealants and restorative materials during childhood. Human biomonitoring studies performed in children have demonstrated exposure patterns comparable to those reported in adults, with transient increases in salivary or urinary BPA concentrations observed shortly after treatment (80, 114). Although most human investigations have focused primarily on exposure assessment, a limited number of studies have examined health-related outcomes in relation to resin-based dental treatment. Relevant evidence derives from secondary analyses of the New England Children's Amalgam Trial. In an analysis of psychosocial outcomes, the parent trial included 534 children aged 6–10 years at baseline, with follow-up psychosocial data available for 434 participants. Greater cumulative treatment with Bis-GMA–based composite restorations was associated with poorer scores on selected child-reported psychosocial measures, whereas changes in parent-reported outcomes were not consistently significant (146). Importantly, although the parent trial involved randomized assignment to restorative treatment strategies, the analysis according to cumulative amount of composite treatment was observational, and BPA biomarkers were not measured. These findings therefore indicate an association with cumulative Bis-GMA–based composite treatment but cannot establish that BPA exposure was responsible for the observed psychosocial outcomes.

A separate immune function sub study from the same trial included 59 children and assessed immune markers before treatment and repeatedly during follow-up of up to five years. Total white blood cell counts and T-cell and neutrophil responsiveness were not appreciably altered in relation to composite treatment levels. Greater changes in B-cell responsiveness were observed among children with greater exposure to Bis-GMA–based composite restorations, whereas a reduction in monocyte responsiveness observed at six months did not persist over longer follow-up (147). Overall, the study did not identify overt alterations in immune function associated with resin-composite treatment. Because the psychosocial and immune-function analyses were derived from the same parent trial, they should be regarded as complementary analyses of a common study population rather than as independent replication.

Evidence concerning endocrine-related outcomes in adults is similarly limited. Gul et al. evaluated 18 healthy men receiving two composite restorations and measured BPA concentrations in saliva and serum together with reproductive hormone levels before treatment and at 1 day and 1, 3, and 5 weeks after restoration placement (148). BPA concentrations did not increase significantly over the observation period, and no significant alterations in serum reproductive hormone levels attributable to BPA were identified. Nevertheless, the small sample size and five-week follow-up substantially limit the ability of this study to detect uncommon effects or to draw conclusions regarding longer-term endocrine outcomes.

Pregnancy represents a potentially susceptible exposure window in the broader BPA literature; however, the available evidence primarily concerns environmental or prenatal BPA exposure rather than BPA specifically attributable to dental treatment (149–151). These studies therefore provide indirect background evidence and should not be interpreted as clinical evidence of dental-source BPA exposure during pregnancy. Longitudinal investigations assessing long-term health outcomes specifically attributable to BPA released from dental materials remain scarce. Collectively, the available human evidence includes both potentially concerning associations and largely null findings; however, it remains insufficient to establish a causal relationship between BPA exposure specifically attributable to dental materials and clinically measurable adverse health outcomes.

To facilitate comparison across the heterogeneous clinical evidence, selected clinically relevant human and biomonitoring studies evaluating BPA exposure associated with dental materials and procedures are summarized in Supplementary Table 1. The table provides a comparative overview of the dental material or procedure investigated, study population, biological matrix, sampling schedule, analytical methodology, reported BPA concentrations or exposure patterns, and principal clinical interpretation.

## 4 Discussion

The evidence synthesized in the present review indicates that the scientific discussion surrounding BPA in dentistry has progressively evolved from documenting its analytical detectability toward understanding the biological and clinical relevance of exposure under contemporary clinical conditions. This evolution reflects the growing recognition that the presence of a chemical compound alone provides only partial information regarding its potential health implications. Indeed, analytical detection, biological availability, systemic exposure, and clinically relevant biological effects represent distinct yet interconnected processes that should not be interpreted interchangeably. Appreciating this distinction is essential for an objective interpretation of the current literature and may explain, at least in part, the contrasting conclusions that have characterized the debate surrounding BPA over the past two decades.

One of the principal findings emerging from the available evidence is that BPA associated with dental materials should be interpreted within a broader biological and environmental framework rather than as an isolated phenomenon (16, 31). Contemporary restorative materials are only one of several potential sources contributing to human exposure, which also includes food-contact materials, consumer products, environmental contamination, and occupational settings (8, 152, 153). Consequently, the contribution of dentistry should be considered within the context of the overall human exposome, where cumulative exposure results from the interaction of multiple sources rather than from a single clinical procedure. This perspective is particularly relevant when interpreting biomonitoring studies, as the concentrations measured in saliva, urine, or other biological matrices inevitably reflect not only dental treatment but also the individual's background environmental exposure (143).

Within this context, the increasing sensitivity of modern analytical techniques has profoundly influenced the interpretation of published evidence. Advances in liquid chromatography coupled with tandem mass spectrometry have enabled the reliable identification of BPA at concentrations that were previously beyond analytical detection (154–156). While this technological progress has undoubtedly improved exposure characterization, it has also widened the gap between analytical detectability and biological interpretation. The ability to identify trace amounts of BPA is not synonymous with clinically relevant exposure, nor does it, in isolation, demonstrate clinically meaningful harm.

Likewise, the absence of a statistically significant difference between pre- and post-treatment BPA concentrations in an individual study should not be interpreted as evidence of equivalence, absence of exposure, or biological safety. Conversely, the absence of demonstrated long-term adverse outcomes associated with dental BPA exposure should not be interpreted as definitive evidence of long-term safety. Rather, the available clinical evidence remains insufficient to establish the long-term systemic health implications of such exposure, particularly because most human investigations have focused on short-term biomonitoring following dental procedures rather than on endocrine, reproductive, metabolic, neurodevelopmental, or other clinically relevant systemic outcomes requiring larger populations and substantially longer follow-up periods (22). Consequently, current evidence is insufficient to establish either a causal relationship between dental BPA exposure and long-term systemic adverse health outcomes or the definitive absence of such effects.

The interpretation of the available evidence is further complicated by the substantial methodological heterogeneity that characterizes this field. Differences in resin formulation, specimen preparation, polymerization protocols, extraction media, ageing procedures, surface-area-to-volume ratios, sampling intervals, and analytical methodologies may substantially influence the amount of BPA detected and limit direct comparison among studies (5, 16, 30, 34). Importantly, some *in vitro* degradation studies employ prolonged immersion, organic solvents, markedly acidic or alkaline conditions, or accelerated ageing procedures specifically designed to challenge polymer stability and investigate potential degradation pathways (70–72). Although these experimental conditions are valuable for mechanistic characterization and worst-case assessment, they do not necessarily reproduce the dynamic oral environment or the magnitude and duration of exposure occurring during routine clinical use. BPA concentrations generated under such conditions should therefore not be directly extrapolated to patient exposure or clinical risk. Analytical variability represents an additional source of uncertainty, as differences in assay sensitivity and selectivity, limits of detection and quantification, sample preparation, biological matrix, and the distinction between unconjugated and total BPA may contribute to divergent results across studies (30, 34). Consequently, apparently conflicting findings may reflect methodological rather than biological variability, and no single experimental BPA concentration can currently be regarded as representative of exposure across resin-based dental materials. Greater methodological standardization and closer alignment of experimental conditions with clinically relevant exposure scenarios are therefore essential for improving the translational value of future research.

Another important consideration is that BPA-derived monomers should not be interpreted as a chemically or biologically homogeneous group. Differences in molecular architecture among Bis-GMA, Bis-DMA, and Bis-EMA translate into distinct hydrolytic stability and degradation behavior, as discussed in the preceding sections (33, 47). This chemical heterogeneity has important implications for evidence interpretation: findings obtained for one monomer, formulation, or experimental condition cannot be generalized to all BPA-derived dental materials. Consequently, clinically meaningful assessment requires product-specific characterization and consideration of the conditions under which degradation and release were evaluated rather than classification based solely on the presence of a BPA-derived molecular scaffold (29).

Taken together, these considerations suggest that the central question is no longer whether BPA can be detected following restorative procedures, but whether the concentrations observed under clinically relevant conditions are sufficient to produce biologically meaningful effects within the broader context of lifelong environmental exposure. Addressing this question requires moving beyond a purely analytical perspective and integrating evidence from materials science, analytical chemistry, toxicology, clinical dentistry, epidemiology, and environmental health. Such an integrated interpretation provides a more balanced understanding of the available evidence and establishes the conceptual framework within which the clinical implications of BPA in contemporary restorative dentistry should be discussed.

The available evidence also suggests that the clinical significance of BPA in restorative dentistry should be interpreted within a broader benefit–risk framework. Resin-based materials have profoundly transformed contemporary dental practice by enabling minimally invasive restorative procedures, preservation of sound dental tissues, exhibiting good biomechanical behavior, improved esthetic outcomes, and predictable long-term clinical performance (49, 157–160). Consequently, the discussion surrounding BPA should not focus exclusively on the presence of a single chemical constituent but rather on the overall biological profile of restorative materials in relation to their well-established therapeutic benefits. This perspective is particularly important because clinical decision-making inevitably requires balancing biological considerations with mechanical reliability, longevity, patient-centered outcomes, and the preservation of oral tissues.

The recent revision of the EFSA TDI also requires careful interpretation when considered in relation to dental exposure. As discussed above, this health-based guidance value was developed for chronic daily oral exposure, whereas most clinical dental studies characterize short-term changes in BPA concentrations in saliva, urine, or plasma during defined sampling periods following a discrete procedure. Consequently, direct numerical comparison between biomonitoring concentrations and the TDI is not methodologically appropriate without reliable estimation of the absorbed dose attributable to the dental procedure, including consideration of toxicokinetics and background environmental exposure. The regulatory interpretation is further complicated by continuing scientific debate regarding the derivation of health-based guidance values for BPA. The German Federal Institute for Risk Assessment (BfR), for example, proposed a health-based guidance value of 200 ng/kg body weight/day, substantially higher than the current EFSA TDI, based on a different interpretation of the toxicological evidence and of the suitability of immunological changes as predictors of adverse effects in humans (161). Scientific divergence between EFSA and the European Medicines Agency (EMA) has likewise concerned the definition of adverse effects, the interpretation of intermediate versus apical endpoints, and the biological relevance of the immunological findings used for risk assessment (162). These differences should not be interpreted as demonstrating either the presence or absence of clinically meaningful harm at exposure levels relevant to dentistry; rather, they illustrate the uncertainty involved in translating experimental toxicological findings into quantitative health-based guidance values. Accordingly, the current EFSA TDI represents an important precautionary regulatory benchmark, but its application to dental exposure requires consideration of exposure pattern, duration, absorbed dose, toxicokinetics, background exposure, and the clinical relevance of the underlying toxicological evidence.

Within this framework, several procedural variables relevant to resin-based restorative treatment can be optimized during routine clinical practice, although their potential implications for BPA availability should be interpreted according to the specific outcome investigated. Adequate light curing, verification of curing-light performance, adherence to manufacturers’ instructions, and appropriate incremental placement when indicated are established principles of restorative practice aimed at achieving adequate polymerization and predictable clinical performance (163). Their potential relevance to BPA exposure is indirect, because improved polymerization may reduce the fraction of residual unreacted resin constituents available for subsequent elution; however, the supporting evidence should not be interpreted as demonstrating a corresponding reduction in salivary BPA concentrations, systemic BPA dose, or BPA-related health outcomes.

Finishing and polishing act through a different mechanism and should not be considered procedures that increase bulk monomer conversion. Rather, these procedures modify the restoration surface and may remove or reduce the superficial resin-rich or oxygen-inhibited layer, while also improving surface characteristics (164, 165). Their potential relevance to BPA exposure therefore concerns the removal of superficial residual material rather than an increase in polymerization throughout the restoration. Although surface treatment has been discussed as a possible means of reducing the immediate availability of residual resin constituents, evidence concerning surface properties or removal of the oxygen-inhibited layer should not, in the absence of direct BPA measurements, be interpreted as demonstrating a reduction in systemic BPA exposure or BPA-related health risk. Accordingly, adequate curing and appropriate finishing and polishing remain important components of good restorative practice, while any potential benefit in reducing BPA availability should be interpreted according to the specific endpoint directly evaluated in the supporting studies.

The growing scientific interest in BPA has also accelerated the development of dental restorative materials formulated without BPA-derived monomers (27, 55). Similar trends have been observed in several industrial sectors, where increasing attention to endocrine-disrupting chemicals has promoted the progressive replacement of BPA in food-contact materials, infant products, reusable containers, and other consumer goods (166–168). These regulatory developments should not be interpreted as direct evidence that BPA released from dental materials causes clinically relevant adverse effects. Rather, they reflect a precautionary public health strategy aimed at reducing unnecessary exposure whenever suitable alternatives are available without compromising product performance or safety.

A similar precautionary perspective has recently been adopted by the World Health Organization in its Guideline on Environmentally Friendly and Less Invasive Oral Health Care. Specifically, Recommendation 7 is a conditional recommendation, based on very-low-certainty evidence, to avoid, whenever possible, the use of fissure sealants and resin-based composites containing BPA derivatives in children, adolescents, and pregnant or breastfeeding women (37). This recommendation should therefore be interpreted within its stated evidentiary limitations and as a precautionary approach rather than as confirmation of a causal association between BPA exposure from dental materials and adverse health effects. Where preventive or restorative treatment is required, consideration of alternative materials should remain indication-specific and should consider their clinical suitability, effectiveness, durability, and overall biological profile. Importantly, Recommendation 7 does not identify dental amalgam as the alternative to BPA-containing resin-based materials; rather, the selection of an alternative should be based on the specific clinical indication and on the availability of an appropriate material for the individual patient (37).

The development of alternative resin-based dental materials has increasingly included formulations in which conventional BPA-derived monomers are replaced by UDMA, aliphatic dimethacrylates, or other monomer architectures (54, 55). Importantly, the use of a non-BPA-derived monomer such as UDMA does not, by itself, establish that the complete commercial formulation contains no BPA or other BPA-derived constituents. Accordingly, claims regarding the absence of BPA or BPA-derived compounds should be interpreted according to the complete material composition and the specific analytical evidence available. Such substitutions, however, involve considerably more than the replacement of a potentially undesirable chemical precursor. Monomer structure and resin-network architecture strongly influence viscosity, polymerization kinetics, degree of conversion, cross-link density, water sorption, degradation behavior, and the resulting mechanical properties of resin-based materials (46, 49). Accordingly, substitution of Bis-GMA or other BPA-derived constituents may alter clinically relevant properties rather than representing a chemically or mechanically neutral modification. Recent investigations of BPA-free resin composites have demonstrated that their mechanical behavior is strongly dependent on formulation and filler characteristics, while wear performance may also differ among alternative resin systems (27, 54, 55). Similarly, the bonding performance of BPA-free adhesive formulations remains dependent on the chemistry of the adhesive system and the characteristics of the dental substrate, reinforcing the need to evaluate alternative formulations according to their complete material-specific performance rather than based on BPA absence alone (169). Thus, although non-BPA-derived monomer systems may provide viable alternatives for restorative applications, their potential biological advantages must be considered together with polymerization behavior, physicochemical stability, mechanical reliability, adhesive performance, and long-term clinical durability.

An equally important consideration is that the toxicological evidence available for many alternative monomers and their degradation products remains less extensive than that available for BPA and established BPA-derived resin systems. Terminology concerning “BPA-free” materials requires careful interpretation. “No added free BPA,” “Bis-GMA-free,” and “free of BPA-derived monomers” describe different compositional characteristics and should not be used interchangeably. Likewise, analytical nondetection of BPA indicates only that BPA was not detected under the specific experimental conditions and within the sensitivity of the analytical method employed; it does not establish its absolute absence from the commercial formulation. Accordingly, the BPA-related profile of a material should be described according to the specific compositional or analytical evidence available rather than inferred from the structure of a single monomer. Moreover, the absence of BPA or BPA-derived monomers does not, by itself, provide information regarding the identity, concentration, release kinetics, degradation behavior, metabolism, or biological activity of the alternative constituents used in the formulation. This distinction is particularly relevant because the biological profile of polymeric dental materials may be influenced not only by parent monomers but also by residual monomers, additives, photoinitiators, oligomers, and products generated during chemical or enzymatic degradation (48). Studies comparing 3D-printed, thermoformed, and conventionally polymerized intraoral materials have identified differences in biocompatibility and chemical stability according to material composition and manufacturing approach (170, 171). More broadly, the evaluation of alternative and BPA-free dental materials should not be based exclusively on the absence of BPA-derived constituents, as their overall performance also depends on other material-specific properties, including the release of inorganic components and their adhesive behavior (172, 173). Moreover, favorable results from short-term cytotoxicity testing cannot be interpreted as comprehensive evidence of systemic biological safety, nor can they exclude endocrine, immunological, genotoxic, or other effects requiring different experimental models, exposure conditions, and longer observation periods. Long-term human exposure and clinical outcome data remain particularly limited for many newer formulations. Future evaluation of BPA-free materials should therefore move beyond substitution-based claims and incorporate comprehensive compositional disclosure, quantitative characterization of leachable and degradation products, clinically relevant ageing and mechanical testing, mechanistic toxicological assessment, and, where appropriate, human biomonitoring and longitudinal clinical investigation.

A broader biomaterials perspective therefore supports the evaluation of BPA within the overall chemical and biological profile of contemporary dental polymers rather than considering a single compound in isolation. Evidence from 3D-printed, thermoformed, and conventionally heat-polymerized intraoral materials reinforces the concept that biological safety is a multidimensional property encompassing material composition, manufacturing and post-processing conditions, chemical stability, degradation and elution behavior, and tissue responses (172–174). Accordingly, the development of BPA-free or alternative polymeric formulations should not, by itself, be considered sufficient to establish an improved biological profile; emerging materials and manufacturing technologies require integrated chemical, mechanical, and biological characterization under clinically relevant conditions.

This broader vision closely reflects the emerging concept of Safe and Sustainable by Design (SSbD), according to which chemical safety, clinical performance, durability, environmental sustainability, and transparency should be considered simultaneously from the earliest stages of biomaterial development (175). Applied to restorative dentistry, this approach moves the scientific debate beyond the isolated issue of BPA and toward a more comprehensive strategy aimed at developing materials that remain effective, durable, biocompatible, and environmentally responsible throughout their clinical use (176). Such a transition appears particularly relevant in view of the increasing emphasis placed on sustainability within healthcare systems and may represent one of the most promising directions for future restorative material research.

Finally, these considerations have different implications for the management of existing restorations and for material selection when new treatment is required. For existing restorations, the presence of BPA-derived constituents alone does not currently constitute a demonstrated health-based indication for the elective replacement of an otherwise clinically satisfactory resin restoration. Such replacement would require an additional operative procedure, removal of further dental tissue, and generation of additional restorative waste without established evidence of a corresponding health benefit. Accordingly, the available BPA-related evidence does not provide a basis for elective replacement of otherwise satisfactory restorations; preservation or repair, when otherwise clinically appropriate, remains consistent with minimally invasive restorative principles (52, 177).

Material selection for a new restoration or preventive procedure represents a separate clinical decision. When treatment is indicated, clinicians may consider material composition alongside the specific clinical indication, mechanical and adhesive requirements, expected durability, patient-related factors, and the overall biological profile of the available materials (178). Where clinically suitable alternatives are available, minimizing unnecessary exposure to BPA-derived constituents may be considered as part of this broader decision-making process, particularly for populations addressed by current precautionary guidance. However, material selection should not be based on the presence or absence of BPA-derived constituents alone, and the available evidence does not establish that choosing a BPA-free material necessarily results in superior clinical or systemic health outcomes.

The present review should be interpreted considering several limitations affecting both the underlying evidence base and the review methodology. The available literature is characterized by substantial methodological heterogeneity with respect to material formulations, experimental and clinical designs, ageing protocols, extraction media, exposure conditions, biological matrices, analytical methods and detection limits, sampling intervals, follow-up periods, and reported outcomes. These differences limit direct quantitative comparisons across studies, preclude a uniform assessment of BPA exposure or its biological effects, and may contribute to apparently conflicting findings. An additional limitation arises from the continuous evolution of commercial dental materials. Manufacturers may modify resin formulations over time, while complete compositional information is not always publicly available. Consequently, products investigated in earlier studies may not fully reflect current formulations, complicating both evidence synthesis and the translation of historical findings to contemporary clinical practice.

The narrative design of the present review also entails methodological limitations. Literature identification and selection involved a degree of author judgment and were not conducted within a prospectively registered systematic-review protocol. The review was restricted to English-language publications for which sufficient full-text information was available for evaluation, which may have resulted in the omission of relevant evidence published in other languages or not accessible in full text. In addition, complete database-specific search histories and formal screening counts were not retained during the original review process and were therefore not reconstructed retrospectively. Accordingly, the present review should be interpreted as a critical narrative synthesis of the available evidence rather than as an exhaustive systematic assessment of all published studies. Nevertheless, the narrative approach enabled the integration of evidence from materials science, analytical chemistry, toxicology, environmental health, regulatory science, and clinical dentistry, providing a multidisciplinary framework for interpreting BPA-related evidence in a clinically meaningful context.

The limitations of the primary literature also identify several priorities for future investigation. Progress in this field will likely depend less on generating additional isolated measurements of BPA detectability and more on developing integrated translational research capable of linking material composition, degradation processes, analytical chemistry, human biomonitoring, toxicological mechanisms, and clinically relevant outcomes (16). Future studies should therefore adopt standardized experimental protocols, harmonized analytical methodologies, and longitudinal clinical designs that better reflect real-world exposure. Equally important will be the systematic evaluation of emerging BPA-free formulations, ensuring that replacement strategies are supported not only by reduced BPA availability but also by robust evidence regarding their chemical stability, biosafety, mechanical reliability, environmental sustainability, and long-term clinical performance. Such an approach would reduce the risk of replacing well-characterized materials with alternative formulations whose biological profiles remain insufficiently understood.

Greater transparency regarding material composition should represent another priority for future research and dental industrial development. More comprehensive disclosure of resin formulations would facilitate independent scientific evaluation, improve reproducibility, strengthen evidence synthesis, and enable clinicians to make more informed decisions when selecting restorative materials. As dental materials research continues to evolve, chemical transparency should be regarded as an integral component of biosafety rather than merely a regulatory requirement.

Ultimately, the evidence currently available supports a balanced and precautionary interpretation of BPA in dentistry. Resin-based materials remain fundamental to contemporary minimally invasive dentistry because of their well-established clinical effectiveness, while the amounts of BPA detected under experimental and clinical conditions should be interpreted within the broader context of cumulative environmental exposure and the limitations of the existing evidence. Importantly, current human data neither establish a causal relationship between dental BPA exposure and clinically relevant systemic adverse outcomes nor provide definitive evidence regarding long-term safety. Accordingly, the scientific debate should progressively move beyond the simple question of whether BPA is detectable and instead focus on improving the overall biological profile of dental materials through rigorous chemical characterization, clinically relevant exposure assessment, and long-term outcome evaluation. Within this evolving framework, minimizing avoidable BPA exposure should be regarded as one component of a broader strategy aimed at improving the biosafety, chemical transparency, environmental sustainability, and clinical performance of dental materials while preserving the substantial therapeutic benefits that adhesive dentistry has brought to modern oral healthcare.

## 5 Conclusions

Growing scientific interest in bisphenol A has substantially improved our understanding of the complex interactions among dental materials, the oral environment, and human exposure while also highlighting the challenges of translating analytical detection and experimental biological activity into clinically meaningful risk assessment. Available human biomonitoring evidence indicates that selected resin-based dental procedures may be followed by short-term changes in BPA concentrations, although the magnitude and temporal pattern vary according to the dental procedure, treatment burden, biological matrix, sampling interval, analytical methodology, and background environmental exposure. In studies reporting post-treatment increases, these changes have generally been observed during the early sampling period and subsequently declined or were no longer significantly different from pretreatment concentrations at later study-specific time points. However, the long-term systemic clinical significance of dental BPA exposure remains incompletely characterized. Importantly, the absence of demonstrated long-term adverse health outcomes should not be interpreted as definitive evidence of long-term safety, particularly because longitudinal studies specifically evaluating clinically relevant systemic outcomes following dental BPA exposure remain limited. Conversely, analytical detection of BPA, evidence of internal exposure, or biological activity demonstrated under experimental conditions does not, in isolation, establish clinically meaningful harm.

Current evidence therefore supports a balanced and precautionary clinical approach that considers the overall biological and clinical performance of dental materials while minimizing avoidable exposure whenever reasonably achievable. Current evidence therefore supports a balanced and precautionary clinical approach that considers the overall biological and clinical performance of dental materials while minimizing avoidable exposure whenever reasonably achievable. Adequate polymerization and appropriate finishing and polishing remain important components of good restorative practice, although their relevance to BPA availability differs according to the mechanism involved and the endpoint investigated. Evidence concerning improved polymerization, removal of superficial residual material, or modification of the oxygen-inhibited layer should not be interpreted as direct evidence of reduced systemic BPA exposure or BPA-related health risk. At the same time, the absence of BPA should not itself be regarded as evidence of a superior biological profile, as alternative monomers and degradation products require equally rigorous chemical and toxicological characterization.

Future progress will require longitudinal and clinically oriented investigations capable of integrating material composition, degradation behavior, human biomonitoring, mechanistic evidence, and clinically relevant systemic outcomes, particularly under repeated or cumulative exposure conditions and in potentially susceptible populations. Greater compositional transparency and rigorous evaluation of emerging BPA-free formulations will also be essential. Such an integrated approach may support the development of dental materials with increasingly well-characterized biological profiles, greater chemical transparency, improved environmental sustainability, and durable clinical performance.
