# Supplementary material for: Bisphenol A in contemporary dentistry: from material chemistry and exposure pathways to potential clinical relevance—a comprehensive narrative review
Source: Front Dent Med. 2026 Oct 6;7:1972627. doi: 10.3389/fdmed.2026.1972627 (PMC13681359; doi:10.3389/fdmed.2026.1972627)
Supplement: Supplementary file 1 [file Table1.docx]

***Supplementary Material***

**Supplementary Table S1.** Selected human studies evaluating BPA exposure, biomonitoring findings, and health-related outcomes associated with resin-based dental materials and procedures.

| Study | Participants | Material/procedure and treatment burden | Biological matrix | BPA fraction measured | Sampling times | Baseline BPA | Post-treatment BPA / main findings | Analytical method | LOD/LOQ & nondetect handling | Urine dilution adjustment | Contamination controls / competing exposures | Main interpretation / limitations |  |
| --- | --- | --- | --- | --- | --- | --- | --- | --- | --- | --- | --- | --- | --- |
| A. Human biomonitoring and exposure studies | | | | | | | | | | | | | |
| Berge et al., 2019 (23) | 20 adults requiring ≥1 polymer-based dental restoration | Polymer-based restorative treatment; mean 3.7 ± 1.9 filling surfaces (range 2–10); mean material weight 0.158 ± 0.067 g | Saliva; urine | Free (unconjugated) BPA in saliva; total BPA in urine after enzymatic deconjugation | Pretreatment; saliva: 10 min, 1 h, 24 h, 1 week; urine: 1 h, 24 h, 1 week | Saliva: 0.11 ng/mL; density-adjusted urine: 1.41 ng/mL | Saliva: 385 ng/mL (10 min), 88.2 (1 h), 1.85 (24 h), 0.25 (1 week). Density-adjusted urine: 1.17 (1 h), 1.53 (24 h), 1.99 (1 week); no significant post-treatment change. | LC/MS; selected saliva samples confirmed independently by LC-MS/MS | LOD 0.1 ng/mL; values <LOD assigned 0.05 ng/mL; LOQ NR | Density-adjusted to reference density 1.016 | Chemical blanks and QC samples analyzed; one probably contaminated baseline saliva sample excluded. Diet and thermal-paper receipt handling evaluated as competing exposures. | Marked early salivary increase followed by rapid decline; no significant corresponding urinary change. Matrix-specific patterns, interindividual variability, and environmental exposure complicate dental-source attribution. |  |
| Chung et al., 2012 (114) | 495 South Korean children aged 8–9 years | Existing resin composites and sealants; groups: 0, 1–5, 6–10, or ≥11 treated surfaces | Urine | NR | Single cross-sectional sample | N/A as longitudinal baseline; 0 treated surfaces served as reference | Children with ≥11 resin-composite surfaces had adjusted urinary BPA of 2.67 μg/g creatinine, significantly higher than children with no restored surfaces; no corresponding increase for 1–10 surfaces. | NR in accessible report | NR | Creatinine-adjusted (μg/g creatinine) | Cross-sectional design; nondental/environmental BPA exposure could not be excluded | Association between high treatment burden and urinary BPA; temporality and dental-source causation cannot be established. |  |
| Cik et al., 2025 (140) | 78 orthodontic patients | Fixed appliances, temporary anchorage devices, removable appliances, or functional appliances | Saliva; plasma | NR | Baseline; 1 day; 1 week; 1 month | Group-specific baseline values reported; no single pooled baseline estimate | Across groups/time points: saliva 0.07–7.28 ng/mL; plasma 0.17–12.51 ng/mL. No consistent significant within-group temporal changes after multiplicity correction. | UPLC-MS/MS | NR | N/A | Baseline/background exposure may contribute; specific contamination-control details NR | BPA was measured in both matrices, but nonsignificant temporal differences do not establish equivalence, absence of exposure, or safety. Non-randomized design and baseline variability limit treatment-specific attribution. |  |
| Han et al., 2012 (115) | 124 age- and sex-matched Korean children: 62 controls and 62 with >4 treated surfaces | Existing dental sealants and/or resin restorations; time since placement unavailable | Saliva | NR | Single cross-sectional sample | N/A as longitudinal baseline; adjusted mean in controls: 0.40 μg/L | Overall range 0.002–8.305 μg/L; adjusted mean 0.92 μg/L in children with treated surfaces vs 0.40 μg/L in controls. | ELISA | NR | N/A | Potential environmental exposure remained possible; timing of placement unavailable | Higher salivary BPA was associated with multiple treated surfaces, but cross-sectional design and unavailable placement timing preclude temporal or causal inference. |  |
| Kingman et al., 2012 (83) | 172 participants; saliva analyzed in 151 and urine in 171 | Placement of resin-based composite restorations; treatment burden varied | Saliva; urine | NR | Pretreatment; <1 h; 1–8 h; 9–30 h | Salivary geometric mean ≈0.43 ng/mL; urinary baseline reported in original study | Salivary geometric mean ≈0.64 ng/mL within 1 h and returned to prerestoration levels within ~8 h. Urinary BPA increased by ~43% relative to prerestoration concentrations at 9–30 h. | LC/MS | NR | NR | NR | Composite placement was associated with short-term, matrix-dependent changes. Salivary and urinary patterns differed; 'transient' should therefore be linked to matrix and sampling interval. |  |
| Maserejian et al., 2016 (80) | 91 children/adolescents aged 3–17 years | Bis-GMA-based composite restorations; mean 1.4 ± 1.0 restored surfaces at first visit and 2.3 ± 1.6 cumulatively | Urine | Total urinary BPA | Pretreatment; ~1 day; 14 days; 6 months | Within-person pretreatment value used as baseline | Mean next-day change +1.71 ± 9.94 ng/mL. One participant with 8 restored surfaces had next-day BPA 81.8 ng/mL. Excluding this observation reduced mean change to +0.87 ± 5.98 ng/mL; the treatment-burden association was attenuated and no longer significant. No association at ~14 days or 6 months. | Online SPE-HPLC-isotope-dilution MS/MS | NR | Specific gravity incorporated in analyses | Information on BPA-related exposures collected; background BPA exposure recognized as an important source of variability | Primary next-day association was sensitive to an influential high-treatment observation and was not present at later follow-up; findings do not support a uniform urinary BPA increase after treatment. |  |
| McKinney et al., 2014 (142) | 1,001 U.S. children aged 6–19 years (NHANES 2003–2004) | Existing occlusal sealants and restorations; material type and placement timing unavailable | Urine | Total urinary BPA | Single cross-sectional assessment | N/A as longitudinal baseline | Children with 7–16 sealants had adjusted geometric mean BPA 25% higher than those with none (95% CI −14% to 82%; P=.23); those with 7–42 restorations had 20% higher BPA than those with none (95% CI −6% to 53%; P=.13). | HPLC-MS/MS (NHANES biomonitoring method) | LOD 0.3 ng/mL; values below LOD included in left-censored Tobit models; sensitivity analyses used LOD/√2 substitution | Urinary creatinine included in adjusted analyses | Cross-sectional NHANES design; material type, placement timing, and nondental BPA sources limit attribution | Population-level associations were not statistically significant and cannot define within-person post-treatment change or temporality. |  |
| Sasaki et al., 2005 (110) | Patients receiving composite resin restorations | Composite resin restoration; multiple commercial resin systems evaluated | Saliva | NR | Before and immediately after restoration placement; post-rinse assessment reported | Material-specific pretreatment values reported | Post-treatment salivary BPA varied by material; mean 32.1 ng/mL and maximum 60.1 ng/mL reported for one tested condition/material group; concentrations decreased after rinsing. | ELISA | NR | N/A | NR | Short-term salivary BPA detection varied by material. Salivary measurements represent local oral exposure and should not be interpreted as systemic dose; results obtained by ELISA are not directly interchangeable with MS-based measurements. |  |
| Vitores-Calero et al., 2025 (125) | 24 patients: 12 Invisalign® and 12 in-office aligner patients | Clear-aligner treatment with composite attachment bonding | Saliva | NR | Baseline; 30 min after attachment bonding; 30 min after aligner placement; 1 week | Invisalign® median 5.03 ppb; group-specific baseline values reported | Invisalign® median 37.3 ppb after attachment bonding, 11.6 ppb after aligner placement, and 7.09 ppb at 1 week; early peak occurred after attachment bonding. | HPLC-MS | NR | N/A | Composite attachment bonding is a competing dental source relative to the aligner itself; other contamination-control details NR | Early peak cannot be attributed to the aligner material alone. Small sample and short follow-up limit generalization. |  |
| B. Human health-outcome studies | | | | | | | | | | | | | |
| Maserejian et al., 2012 (146) | 534 children aged 6–10 years at baseline; 434 with psychosocial follow-up data | New England Children’s Amalgam Trial; cumulative Bis-GMA-based composite treatment evaluated in treatment-level analyses | N/A (health-outcome analysis) | Not measured | Psychosocial outcomes over 5-year follow-up | N/A | Greater cumulative Bis-GMA-based composite treatment was associated with poorer scores on selected child-reported psychosocial measures; parent-reported changes were not consistently significant. | N/A | N/A | N/A | BPA biomarkers were not measured; cumulative treatment-amount analysis was observational despite randomized assignment in the parent trial | Association concerns composite treatment, not measured BPA exposure. Findings cannot establish BPA-specific causation. |  |
| Maserejian et al., 2014 (147) | 59 children from the New England Children’s Amalgam Trial immune-function substudy | Bis-GMA-based flowable sealants, Bis-GMA-based restorations, and UDMA-based compomer restorations; treatment levels assessed longitudinally | N/A (immune-function outcomes) | Not measured | Pretreatment and up to 5 post-treatment assessments through 5 years | N/A | No appreciable changes in total WBC counts or T-cell/neutrophil responsiveness. Greater B-cell responsiveness changes occurred with greater Bis-GMA composite exposure; reduced monocyte responsiveness at 6 months did not persist over longer follow-up. | N/A | N/A | N/A | Same parent cohort as psychosocial analysis; BPA biomarkers not measured | No overt immune-function alterations overall; selected cellular findings were inconsistent or transient. This is complementary evidence from the same parent cohort, not independent replication. |  |
| Gul et al., 2021 (148) | 18 healthy men | Two composite restorations per participant | Saliva; serum | BPA measured; fraction not specified in the report used here | Pretreatment; 1 day; 1 week; 3 weeks; 5 weeks | Baseline saliva and serum BPA measured | BPA was not significantly elevated over time in saliva or serum. Serum reproductive hormone levels showed no significant BPA-attributable alterations under the study conditions. | HPLC for BPA; radioimmunoassay kits for reproductive hormones | NR | N/A | Small sample; background BPA exposure cannot be excluded; contamination-control details NR | Nonsignificant findings do not demonstrate equivalence or long-term safety. Sample size (n=18) and 5-week follow-up limit inference regarding uncommon or longer-term endocrine effects. |  |

**Abbreviations:** BPA, bisphenol A; ELISA, enzyme-linked immunosorbent assay; HPLC, high-performance liquid chromatography; LC/MS, liquid chromatography–mass spectrometry; LC-MS/MS, liquid chromatography–tandem mass spectrometry; UPLC-MS/MS, ultra-performance liquid chromatography–tandem mass spectrometry; SPE, solid-phase extraction; NHANES, National Health and Nutrition Examination Survey; LOD, limit of detection; LOQ, limit of quantification; NR, not reported; N/A, not applicable; WBC, white blood cell; UDMA, urethane dimethacrylate.

***Note:*** BPA concentrations are reported according to the biological matrix, units, and analytical approach used in the original studies. Salivary, urinary, serum, and plasma concentrations are not directly interchangeable and should not be interpreted as equivalent to estimated BPA intake or health-based guidance values expressed as ng/kg body weight/day. A nonsignificant difference should not be interpreted as evidence of equivalence, absence of exposure, or biological safety. NR indicates information not reported or not sufficiently specified in the source.
